# Effect of*Bacillus velezensis* JC-K3 on Endophytic Bacterial and Fungal Diversity in Wheat Under Salt Stress

**DOI:** 10.3389/fmicb.2021.802054

**Published:** 2021-12-20

**Authors:** Chao Ji, Xiaohui Wang, Xin Song, Qisheng Zhou, Chaohui Li, Zhizhang Chen, Qixiong Gao, Huying Li, Jintai Li, Pengcheng Zhang, Hui Cao

**Affiliations:** ^1^Key Laboratory of Biochemistry and Molecular Biology in University of Shandong Province, Weifang University, Weifang, China; ^2^College of Biological and Agricultural Engineering, Weifang University, Weifang, China; ^3^College of Forestry, Shandong Agriculture University, Taian, China; ^4^Key Laboratory of National Forestry and Grassland Administration on Silviculture of the Lower Yellow River, Shandong Agricultural University, Taian, China; ^5^Ministry of Agriculture Key Laboratory of Seaweed Fertilizers, Qingdao, China; ^6^Animal Husbandry and Veterinary Service Center of Xintai City, Taian, China; ^7^College of Foreign Languages, Weifang University, Weifang, China

**Keywords:** *Bacillus velezensis*, endophytes, microbial inoculants, plant growth-promoting rhizobacteria, endophytic bacteria JC-K3 in salt tolerance, induced systemic tolerance

## Abstract

Plant growth-promoting bacteria (PGPB) can effectively reduce salt damage in plants. Currently, there are many studies on the effects of PGPB on the microbial community structure of rhizosphere soil under salt stress, but fewer studies on the community structure of endophytic bacteria and fungi. We propose that inoculation of endophytic bacteria into the rhizosphere of plants can significantly affect the microbial community structure of the plant’s above-ground and underground parts, which may be the cause of the plant’s “Induced Systemic Tolerance.” The isolated endophytes were re-inoculated into the rhizosphere under salinity stress. We found that, compared with the control group, inoculation with endophytic *Bacillus velezensis* JC-K3 not only increased the accumulation of wheat biomass, but also increased the content of soluble sugar and chlorophyll in wheat, and reduced the absorption of Na in wheat shoots and leaves. The abundance of bacterial communities in shoots and leaves increased and the abundance of fungal communities decreased after inoculation with JC-K3. The fungal community richness of wheat rhizosphere soil was significantly increased. The diversity of bacterial communities in shoots and leaves increased, and the richness of fungal communities decreased. JC-K3 strain improved wheat’s biomass accumulation ability, osmotic adjustment ability, and ion selective absorption ability. In addition, JC-K3 significantly altered the diversity and abundance of endophytic and rhizosphere microorganisms in wheat. PGPB can effectively reduce plant salt damage. At present, there are many studies on the effect of PGPB on the microbial community structure in rhizosphere soil under salt stress, but there are few studies on the community structure changes of endophytic bacteria and fungi in plants.

## Introduction

Soil salinity is one of the main manifestations of global land degradation. At present, at least 20% of the cultivated land in the world is threatened by salinization to varying degrees (Zhu et al., 2011). Although plants can produce a certain amount of yield in saline soil habitats, most crops and trees have low salt tolerance. The yield is reduced by 70% in wheat, corn, rice, barley, and other major food crops under salt stress (Acquaah, 2007).

Salt stress has many effects on plant growth and development. The duration and severity of stress may cause varying degrees of damage to the plant. For example, initial osmotic stress, oxidative stress, and ionic toxicity may cause damage to plant cell membrane structures, nutritional imbalance, reduced antioxidant enzyme activity, and reduced photosynthesis (Rozema and Flowers, 2008; James et al., 2011). With the intake of a large amount of Na^+^ and Cl^−^, the cell ion balance and physiological balance are broken, the water absorption capacity of the plant root is reduced, and the water in the leaves is reduced (Afrasyab et al., 2010). Plants have difficulty in absorbing K^+^, leading to nutrient deficiency, reduced productivity, and even death (James et al., 2011).

Endophytes are microorganisms that form symptomless infections in healthy plants tissues (Carroll et al., 1986; Carroll, 1988). Recent studies have shown that endophytic bacteria can improve the adaptability of plants to adversity (Glick, 2012). These beneficial endophytes are called plant growth-promoting bacteria (PGPB), and they are mostly found in soil, rhizosphere, and leaves (Glick, 2005; Li et al., 2021). It is currently known that certain characteristics of PGPB can improve plant salt tolerance, such as promoting plant nutrition, especially absorption of mineral elements, improving plant salt and alkali resistance, increasing water absorption, increasing osmotic adjustment ability, and changing plants hormone levels. It has important roles in promoting the development and growth of roots, shoots, and leaves of rhizosphere plants, controlling diseases, and regulating the structure of plant microbial communities in the rhizosphere (Kaushal and Wani, 2016; Etesami and Beattie, 2017, 2018; Cordovez et al., 2019). *Bacillus velezensis* belongs to the genus *Bacillus* (Pinchuk et al., 2002) and was isolated much later than that of *Bacillus subtilis* and *Bacillus amyloliquefaciens* (Priest et al., 1987), but its phenotype and phylogeny relationship are closely related (Wang et al., 2008). *Bacillus velezensis* can survive in a medium with a salt concentration of 12%, a temperature range of 15–45°C, and a pH range of 5–10 (Ruiz-Garcia et al., 2005). Numerous studies have shown that *Bacillus velezensis* can produce 3-indoleacetic acid (IAA), ammonia (NH_3_; Meng et al., 2016), siderophore (Sang et al., 2017), bacteriostatic substances (Fan et al., 2017), and lipopeptide antibiotics (Kaki et al., 2013), which can promote plant root development, nutrient absorption, promote plant growth, and increase plant resistance.

In this study, a strain of endophytic *Bacillus velezensis* JC-K3 was isolated from wheat grown on saline soils. This strain not only has strong salt tolerance and alkali resistance, but also produce IAA, siderophore, proline, soluble sugar, protease, cellulase, and glucanase, which have the potential to improve plant salt tolerance and induce systemic tolerance (IST). We investigated the effects of this strain on wheat growth, osmotic regulation, and photosynthetic parameters. In addition, we used 16S rDNA and ITS amplicon sequencing to determine the effects of this strain on endophytic bacteria and endophytic fungi in wheat roots, shoots, and leaves, and the bacterial and fungal community structure in rhizosphere soil. We hypothesized that the addition of the ubiquitous *Bacillus* species to the germinated wheat wound alters the properties of the endophytic bacterial community, but this wound depend on the identity of the species added as different species affect plants in different ways (Gadhave et al., 2016). At the same time, *Bacillus velezensis* has antagonistic properties, which can also significantly affect wheat endophytes and fungal community structure and rhizosphere soil. However, understanding the effects of PGPB on endophytic bacteria, endophytic fungi, rhizosphere bacteria, and rhizosphere fungi community structure in wheat may help us to better understand the complex network of interactions between hosts and microorganisms.

## Materials and Methods

### Isolation of Bacteria

Wheat samples were collected from the Yellow River delta of Shandong province, China (118°49'15"E, 37°24'31"N). Briefly, to isolate the bacterium, roots (5 g, fresh weight) were thoroughly disinfected, homogenized in 0.5X Phosphate buffered Saline (PBS; 9 ml), and serially diluted to 10^−7^ in sterile Nutrient Agar (NA) medium with 4% NaCl concentration, culture in 30°C incubator for 48–72h (Jha et al., 2012). The bacterium was subcultured twice. Finally, the isolates were streaked onto the Nutrient Agar (NA) medium. Glycerol stock solution (30% v/v) of the isolate was prepared and stored at −80°C for further use.

### Stress Tolerance Studies

The tolerance of the selected isolate towards abiotic stressors pH, and salinity was studied. Salt tolerance (2, 4, 6, 8, 10, and 12% NaCl, w/v) was tested on DF agar medium supplemented with 1-aminocyclopropane-1-carboxylate (ACC; 3 mmol l^−1^; Dworkin and Foster, 1958). The strain was streaked on the solid-agar medium and visualized for the growth following incubation at 30°C for 3 days. To measure pH, 100 μl of overnight grown cultures (10^7^ CFU ml^−1^) was added to tryptic soya broth and pH of various ranges (5.0, 6.0, 7.0, 8.0, 9.0, and 10.0) was maintained by 2 mol l^−1^ NaOH and 1 mol l^−1^ HCl using the pH meter (pH FE20, Mettler Toledo, Switzerland). After 3 days, culture pellet was suspended in 2 ml of sterile water, and optical density (OD) was determined at 600 nm in a UV-Visible spectrometer (TU1810, Beijing Purkinje General Instrument Co. Ltd., China) to test the pH tolerance (Singh and Jha, 2017). Each culture was inoculated in triplicate sets.

### Biochemical Characterization and Identification of Strain JC-K3

According to standard protocols for test strain, physiological and biochemical tests, such as Gram stain, starch agar, IMViC (indole, methyl red, Voges-Proskauer, citrate utilization test), and catalase were utilized (Prescott and Harley, 1999). As well, the BIOLOG identification syshoot (BIOLOG MicrostationTM, Biolog Inc., Hayward, CA, United States) for biochemical testing using different carbon sources was used. Inoculation of strains into 71 carbon sources and 23 chemical susceptibility assays were performed according to the BIOLOG manufacturer’s instructions.

To identify the bacterium at the molecular level, 16S rRNA gene was amplified by PCR using standard method (Singh et al., 2015). Universal primers 27F (5'-AGAGTTTGATCMTGGCTCAG-3') and 1492R (5'-TACGGYTACCTTGTTACGACT-3') were used to amplify 16S rRNA gene sequences *via* PCR (Lane, 1991). A sequence comparison between the obtained 16S rRNA sequences and sequences in NCBI was then performed. The pairwise evolutionary distance between 16S rRNA sequence of the test strain and related bacterial strains was calculated, and a phylogenetic tree was constructed by the Neighbor-Joining method using MEGA software (version 7.0.14; Tamura et al., 2011). Bootstrapping of 1,000 replicates was used to cluster the associated taxa.

### Screening for Plant Growth Promoting Attributes

A Salkowski analysis was used to measure the IAA content in the strain after a 48 h incubation in liquid culture containing L-tryptophan (0.5 mg ml^−1^; Moses et al., 2015). According to the method of Singh and Jha (2017), the strain was inoculated into 15 ml Tryptic soy broth medium and cultured for 24 h. The cells were harvested by centrifugation, washed with 0.1 M Tris-HCl (pH 7.6). We added 7.5 ml DF medium (3 mmol ACC as the only nitrogen source) and cultured overnight at 30°C. The bacterial cells were placed in a shaking water bath at 200 rpm and 30°C to induce ACC deaminase for 24 h. According to the protocol of Penrose and Glick (2003), ACC deaminase activity was determined by measuring the amount of α-ketobutyric acid produced by the hydrolytic cleavage of ACC. The ACC deaminase activity of the strain was obtained by comparing the absorbance of the test sample with a standard curve of pure α-ketobutyrate (KB) and measuring the amount of KB at 540 nm. Test of phosphate solubilization was performed in National Botanical Research Institute’s Phosphate (NBRIP) medium supplemented with insoluble tricalcium phosphate and quantified as per the standard protocol (Mehta and Nautiyal, 2001). The siderophore levels in the strain were determined with chrome azurol S (Schwyn and Neilands, 1987). The glucanase activity of the strain was determined using yeast glucan as a substrate (Perez et al., 2002). The protease activity of the strain was determined by measuring the quantity of tyrosine released from casein hydrolysis in the reaction mixture (Bian et al., 2009). The cellulase activity of the strain was determined using sodium carboxymethylcellulose as a substrate (Miller, 1959). The method of Bates et al. (1973) was used in combination with spectrophotometry at 520 nm to determine proline production by the strain.

### Experimental Description

Effect of the bacterial isolate JC-K3 on the growth of wheat plant (*Triticum aestivum* L.) under salinity stress was tested in a controlled environment of plant growth chamber. The soil used for potted plants was 0–20 cm in wheat fields in the Yellow River delta (118°41'07"E, 37°17'17”N; Dongying city, Shandong, China) in October, 2018. The soil is brought back to the greenhouse, broken, and mixed in a 0.5 cm sieve (the number of culturable bacteria and fungi in soil was 1.36 × 10^4^ and 2.14 × 10^3^ CFU g^−1^ dry weight of soil, respectively). Soil and water were mixed in 1:5 ratio; pH and electrical conductivity (EC) were measured with pH meter and conductivity meter (Mettler Toledo, Switzerland). pH 8.329; EC 752 μs cm^−1^; physico-chemical characteristics of soil used in pot were as follows: Organic matter 23.51 g kg^−1^; Total N 1.072 g kg^−1^; Olsen-P 0.0104 g kg^−1^; K^+^ 0.6782 g kg^−1^; Na^+^ 1.0162 g kg^−1^; Ca^2+^ 0.2386 g kg^−1^; and Mg^2+^ 0.5081 g kg^−1^.

Preparation of bacterial inoculum (OD 0.15) and seed treatment was performed according to Penrose and Glick (2003). Wheat was placed in 70% ethanol and 2% sodium hypochlorite solution for 3 min each for surface disinfection. After disinfection, the seeds are thoroughly washed with sterile water to remove all traces of sodium hypochlorite. The surface-sterilized seeds of wheat were kept in the bacterial suspension for 1 h. Surface sterilized seeds treated with 0.03 mol l^−1^ MgSO_4_ instead of bacterial suspension served as control (Singh and Jha, 2017). Twelve bacterized seeds were sown in each plastic pot (20 cm in height, 14 cm in diameter) filled with soil (1.2 KG) and grown with 16:8 photoperiods for 28 days after seed germination at 16–24°C.

### Effect of JC-K3 on Plant Growth Under NaCl Stress Conditions

For measuring growth (root length/plant height) and biomass (fresh/dry weight), six randomly selected plants from each replicate were used. Leaf soluble sugar was extracted from boiling water and quantified *via* the method of Thomas (1977). The proline content was determined according to the method described by Bates et al. (1973), wherein valine was extracted with 3% sulfosalicylic acid and filtered. An aliquot of the filtrate was supplemented with 1 ml ninhydrin and glacial acetic acid reagent. The mixture was boiled for 1 h and placed on ice to stop the reaction, following which absorbance of the sample was measured at 520 nm using a UV spectrophotometer (TU1810, Beijing Purkinje General Instrument Co. Ltd., China).

The method of Moran and Porath (1980) was used to estimate the leaf chlorophyll content. A fresh leaf sample of 0.5 g was taken, extracted with 80% acetone, and centrifuged at 9,000 *g* for 10 min at 4°C. The absorbance of the collected supernatant at 645 and 663 nm was measured using a UV-visible spectrometer. The method for calculating the total chlorophyll content is as follows:


$$\mathrm{Chlorophyll}=\left[8.02\times A_{633}\right]-\left[20.02\times A_{645}\right]$$

Plants were treated according to Singh and Jha (2017). Briefly, the plants were washed in ice-cold 20 mmol l^−1^ CaCl_2_ for 8–10 min, repeated twice, and then washed with ultra pure water 5–6 times. Roots and shoots were separated and oven dried at 70°C for 48 h. One gram of plant tissue was ground in liquid N_2_ and digested in a mixture of 30% H_2_O_2_, 65% HNO_3_, and deionized water at a ratio of 1:1:1 at 120°C for 2 h, with a final volume of 12 ml. The contents of Na^+^ and K^+^ were measured *via* inductively coupled plasma optical emission spectroscopy (ICP, Thermo Scientific™ iCAP™ 7000 Plus, United States; Singh and Jha, 2016).

### Total Bacterial DNA Extraction

Non-rhizosphere soil was removed by gentle shaking, leaving behind only the rhizosphere soil and soil that was still adhered to the roots was considered rhizosphere soil (Smalla et al., 1993). Surface sterilization of collected plant material was performed as described in Wemheuer et al. (2016). The plant material was immersed in 37% formaldehyde for 3 min and rinsed twice with sterile water. Samples were further rinsed with DNA-Exitusplus for 30 s, followed by three washes with sterile water. To confirm the success of the surface sterilization, 100 μl aliquots of the water used in the final washing step were plated on common laboratory media plates. The plates were incubated in the dark at 25°C for at least 2 weeks. The autoclaved mortar and pestle were used to grind the surface-sterilized roots, shoots, and leaves into fine powder in liquid nitrogen, respectively. Ground tissue powder aliquots were subsequently stored at −20°C until DNA extraction (Wemheuer et al., 2017).

To minimize DNA extraction bias, DNA was extracted in quadruplicate from the rhizosphere soil, root, shoot, and leaf samples (Beckers et al., 2017). Microbial DNA was extracted from two treatments using an E.Z.N.A. Soil DNA Kit (Omega Bio-tek, Norcross, GA, United States), according to the manufacturer’s protocol.

V3–V4 region of the bacterial 16S ribosomal RNA gene was amplified by PCR using the following primers: 338F, 5'-barcode-ACTCCTACGGGAGGCAGCA-3' and 806R, 5'-GGACTACHVGGGTWTCTAAT-3', where the barcode is an eight-base sequence unique to each sample. The fungal rDNA-ITS genes were amplified by PCR using primers ITS1F (5'-barcode-CTTGGTCATTTAGAGGAAGTAA-3')/2043R (5'-GCTGCGTTCTTCATCGATGC-3'). PCR was performed in triplicate in 20-μl reaction mixtures containing 4 μl of 5× FastPfu Buffer, 2 μl of 2.5 mmol dNTPs, 0.8 μl of each primer (5 μmol), 0.4 μl FastPfu Polymerase, 10 ng template DNA, and adding ddH_2_O to a final volume of 20 μl.

### Illumina MiSeq Sequencing

Amplicons were extracted from 2% agarose gels and purified using an AxyPrep DNA Gel Extraction Kit (Axygen Biosciences, Union City, CA, United States), according to the manufacturer’s instructions, and quantified using QuantiFluor-ST (Promega, Madison, WI, United States). Purified amplicons were pooled in equimolar amounts and subjected to paired-end sequencing (2,250) on an Illumina MiSeq instrument according to standard protocols. The raw reads were deposited in the NCBI Sequence Read Archive database (accession number: PRJNA642335 and PRJNA643390).

### Processing of Illumina MiSeq Sequencing Data

Raw fastq files were de-multiplexed and quality-filtered using QIIME (version 1.9.1) with the following criteria: (i) 300-bp reads were truncated at any site with an average quality score of less than 20 over a 50-bp sliding window, discarding truncated reads that were shorter than 50 bp; (ii) exact barcode matching, two nucleotide mismatches in primer matching, and reads containing ambiguous characters were removed; and (iii) only sequences that overlapped longer than 10 bp were assembled according to their overlap sequence. Reads that could not be assembled were discarded.

Operational taxonomic units (OTUs) were clustered with a 97% similarity cutoff using UPARSE (version 7.1, http://drive5.com/uparse/), and chimeric sequences were identified and removed using UCHIME. The taxonomy of each 16S rRNA gene sequence was analyzed using the RDP Classifier^1^ against the Silva (SSU123) 16S rRNA database, using a confidence threshold of 70% (Amato et al., 2013). The taxonomy of each ITS rDNA gene sequence was analyzed with the RDP Classifier against the UNITE 7.0/ITS database (Abarenkov et al., 2010) using a confidence threshold of 70%.

### Statistical Analyses

Data analysis was performed using IBM SPSS 19.0. Plant parameters were in accordance with normal distribution; Student’s *t* test was used for parameter differences among plant parameters (*p* < 0.05). Sequences from chloroplasts were also removed before taxonomic classification. R v.2.15.2 (R Foundation for Statistical Computing) was used for Principal co-ordinates analysis (PCoA).

## Results

### Isolation and Primary Characterization of Bacteria

Eight isolates were identified based on morphological differences in the colonies. According to the pH and salt tolerance of the strain, six strains could tolerate 5–10 pH stress, but only three strains could tolerate 8% NaCl (w/v), and a JC-K3 strain with higher tolerance (12% NaCl, w/v) was selected for further study. JC-K3 was inoculated on NA medium and incubated at 28°C for 24 h. The bacteria are rod-shaped, the colonies are milky white, round, slightly convex, and the edges were neat. In terms of physiological and biochemical properties, JC-K3 can hydrolyze gelatin, starch, glyceryl tributyrate, aescin, and casein but not Tween 80. It has oxidase and catalase activity, but does not have arginine dihydrolytic enzyme activity. Nitrate reduction, citric acid utilization, V-P test, and 20°C growth test were all positive. By contrast, H_2_S production, anaerobic growth test, and 50°C growth test were negative. JC-K3 can produce acids from carbon sources, such as glycerin, D-arabinose, L-arabinose, D-robitose, D-xylose, L-xylose and adonitol, D-galactose, D-glucose, D-fructose, D-mannose, L-xylose, inositol, mannitol, sorbitol, alpha-methyl-D-glucoside, N-acetyl glucosamine, esculin, salicin, D-cellobiose, D-lactose, D-maltose, D-melibiose, D-sucrose, D-raffinose, starch, glycogen, and D-lyxose. The salt tolerance of this strain is 12%, and the optimal salt concentration is 4% NaCl. The strain can tolerate pH 5–10.

The 16S rRNA gene sequence of JC-K3 has been submitted to NCBI for Blast analysis. Using a bootstrap analysis of 1,000 datasets, MEGA v. 5.0 was plotted. Phylogenetic tree of JC-K3 sequence and model strain sequence in MEGA v. 5.0. JC-K3 has close homology with *Bacillus velezensis* (Supplementary Figure S1). The 16S rRNA gene sequence of JC-K3 was deposited in the NCBI database as the accession number is: MT605169.

### Plant Growth Promoting Attributes

JC-K3 produced an IAA concentration of 25.48 ± 4.17 μg ml^−1^ after 72 h of bacterial growth, while ACC deaminase activity was 18.10 ± 0.97 μmol (mg h)^−1^ after 48 h of bacterial growth. We noticed that when the strain was inoculated into the NBRIP medium, a transparent circle was formed around the colony. This indicates the solubilization activity of phosphate. When quantifying the solubility of phosphate, it can dissolve phosphorus to a concentration of 78.35 ± 1.84 mg l^−1^ after 72 h of bacterial growth. The appearance of the orange-halo area on CAS-agar plate is considered a positive factor for siderophore carrier. Using desferrioxamine mesylate as a standard compound, the siderophore content in the culture filtrate was 3.76 ± 0.24 μg ml^−1^. It is worth to note that JC-K3 also synthesized glucanase (72 h, 246.76 ± 24.37 μg ml^−1^), protease (72 h, 294.61 ± 15.32 μg ml^−1^), and cellulase (72 h, 40.36 ± 2.67 μg ml^−1^) as well as proline production. In addition, JC-K3 produced proline (72 h, 28.68 ± 0.42 μg ml^−1^; Table 1).

**Table 1:** Plant growth promoting traits of JC-K3.

Plant growth promoting traits	Activity
Salt tolerance	12%
pH tolerance	5–10
IAA production	25.48 ± 4.17 μg ml^−1^
ACC deaminase production	18.10 ± 0.97 μmol (mg·h)^−1^
Phosphate solubilization	78.35 ± 1.84 mg l^−1^
Siderophore production	3.76 ± 0.24 μg ml^−1^
Glucanase	246.76 ± 24.37 u ml^−1^
Protease	294.61 ± 15.32 u ml^−1^
Cellulase	40.36 ± 2.67 u ml^−1^
Proline production	28.68 ± 0.42 μg ml^−1^

Values are mean values ± SDs. Non-inoculated bacteria were used as negative control. + positive, − negative.

### Effect of JC-K3 on Plant Growth Under NaCl Stress Conditions

**Figure 1:**
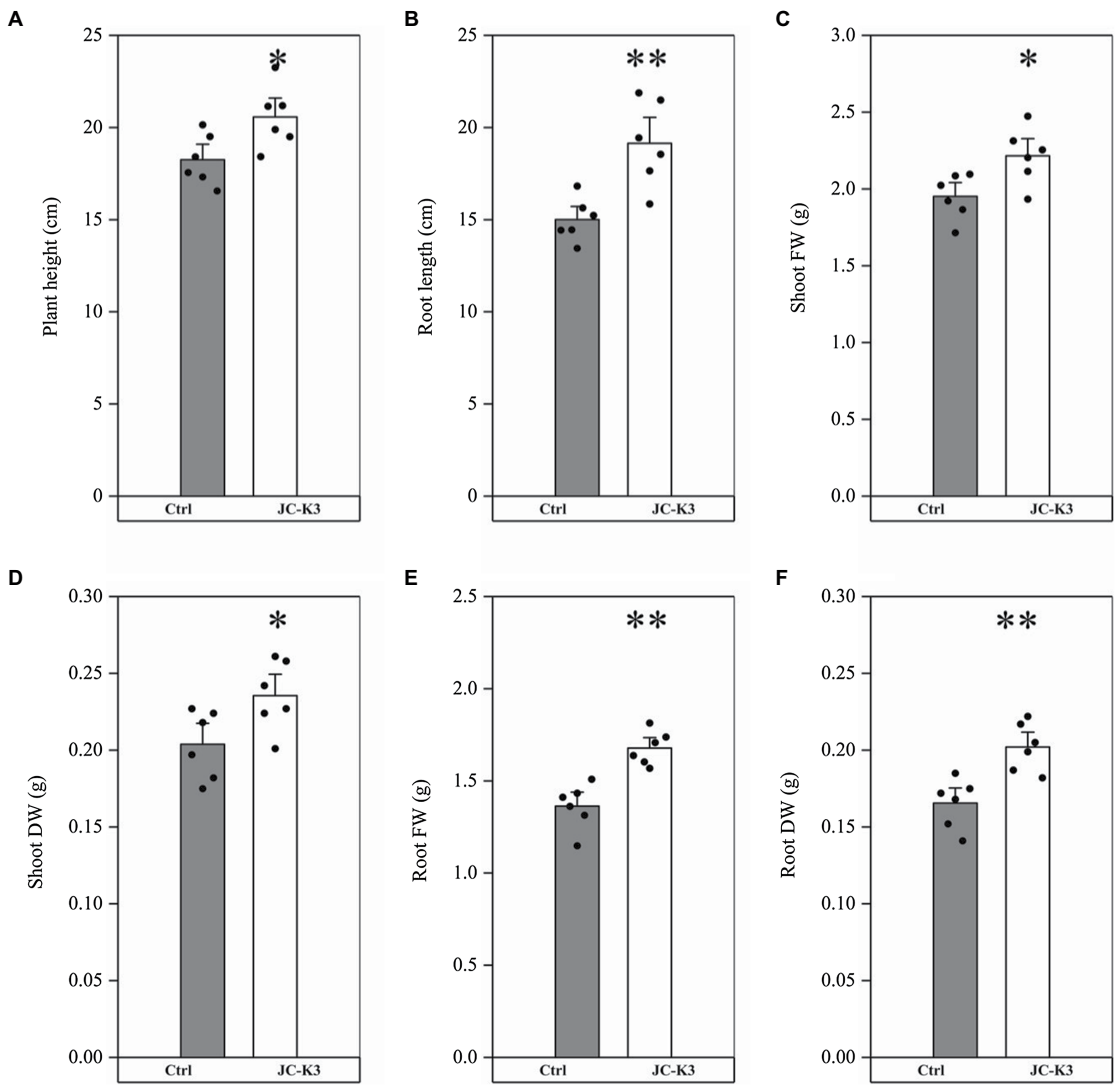
Effect of inoculation of isolate JC-K3 on plant growth and biomass content; **(A)** Plant height, **(B)** Root length, **(C)** Shoot fresh weight, **(D)** Shoot dry weight, **(E)** Root fresh weight, and **(F)** Root dry weight. Each value is the average of six replicates. The error bar represents the SE, the average of six replicates. ^*^ on the bar chart of each treatment indicates the significant difference between the control group and the treatment group, ^*^p < 0.05; ^**^p < 0.01 (Student’s *t*-test). The black and white columns represent the control (Ctrl) and JC-K3 inoculated plants, respectively.

*Bacillus velezensis* JC-K3 enhanced the shoot and root growth of wheat plants under the tested salinity stress. Compared with the control, the plant height of wheat inoculated with JC-K3 increased by 12.69% (*p* < 0.05; Figure 1A), and the root length increased by 27.59% (*p* < 0.01; Figure 1B); the fresh weight of wheat shoot increased by 13.55% (*p* < 0.05; Figure 1C), while the dry weight of shoot increased by 15.54% (*p* < 0.05; Figure 1D); the fresh weight of wheat roots inoculated with JC-K3 increased by 23.14% (*p* < 0.01; Figure 1E), and the dry weight of roots increased by 22.05% (*p* < 0.01; Figure 1F).

**Figure 2:**
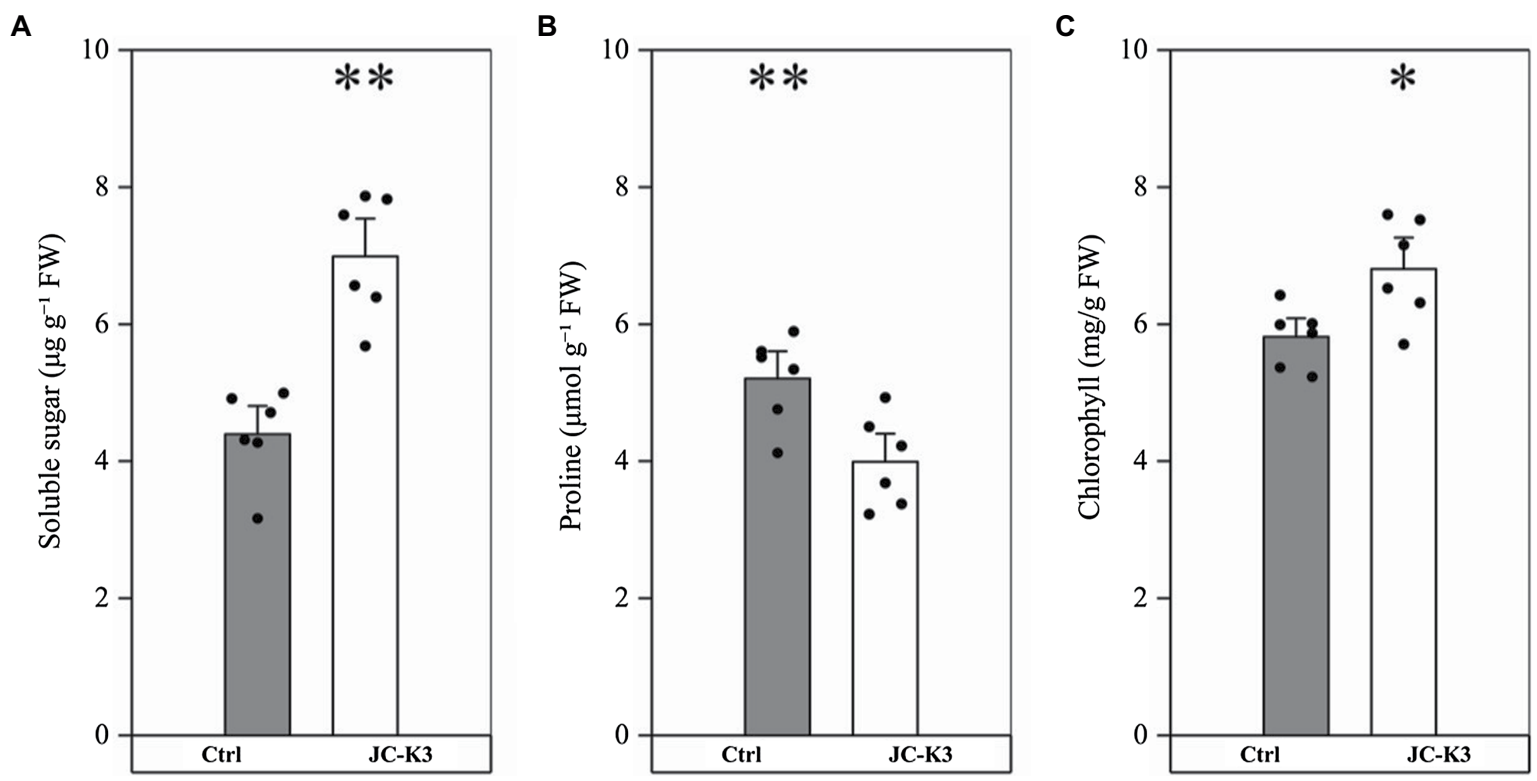
Effect of inoculation isolate JC-K3 on **(A)** total soluble sugar (TSS), **(B)** proline, and **(C)** total chlorophyll content. Each value is the average of six replicates. The error bar represents the SE the average of six replicates. ^*^ on the bar chart of each treatment indicates the significant difference between the control group and the treatment group, ^*^p < 0.05; ^**^p < 0.01 (Student’s *t*-test). The black and white columns represent the control (Ctrl) and JC-K3 inoculated plants, respectively.

The soluble sugar content in wheat inoculated with JC-K3 strain was 6.99 ± 0.82 μg g^−1^ FW, which was 58.86% (*p* < 0.01, Figure 2A) higher than that in the control group (4.40 ± 0.61 μg g^−1^ FW). As can be seen from Figure 2B, the proline content in wheat inoculated with JC-K3 was significantly decreased (23.34%, *p* < 0.01) when compared with the control group. A significant increase in chlorophyll a content was recorded in plants inoculated with JC-K, which was an increase of 17.00% compared to the uninoculated plants (*p* < 0.05; Figure 2C).

**Figure 3:**
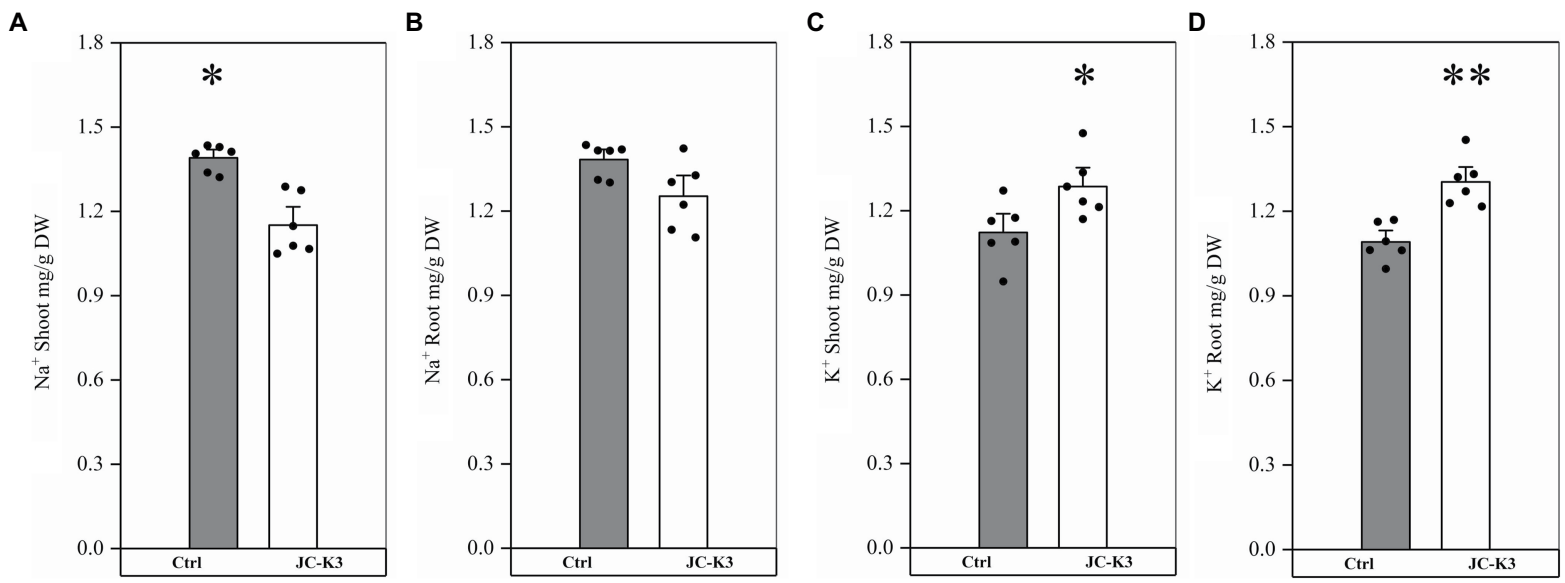
Effect of inoculation of isolate JC-K3 on ionic uptake by plants. **(A)** Shoot Na^+^, **(B)** Root Na^+^, **(C)** Shoot K^+^, and **(D)** Root K^+^. Each value is the average of six replicates. The error bar represents the SE the average of six replicates. ^*^ on the bar chart of each treatment indicates the significant difference between the control group and the treatment group, ^*^p < 0.05; ^**^p < 0.01 (Student’s *t*-test). The black and white columns represent the control (Ctrl) and JC-K3 inoculated plants, respectively.

In order to study the role of PGPB in mitigating NaCl stress, the plant ion balance was fine-tuned. Inductively coupled plasma optical emission spectroscopy (ICP) was used to fine-tune the ion balance of the plant, especially the Na^+^/K^+^ ratio. Ion analysis showed that the Na^+^ content in the shoots and roots of the inoculated test strain JC-K3 decreased, while the K^+^ content increased. Compared with the control, Na^+^ in wheat stalk was reduced by 18.10% (*p* < 0.05; Figure 3A), while Na^+^ in the root was reduced by 9.43% (Figure 3B). Similarly, K^+^ in wheat stalk increased by 16.86% (*p* < 0.05; Figure 3C), while K^+^ in the root increased by 19.51% (*p* < 0.01; Figure 3D) when compared with the control.

### Effects of JC-K3 Strain on Rhizosphere Microbial and Endophyte Community Structure in Wheat

Root, shoot, leaf, and rhizosphere soil samples of the control (CK) and wheat inoculated with JC-K3 strains were used to analyze their community structure. A total of 1,236,843 16S rDNA sequences of V3 and V4 regions were obtained from 24 samples, with an average of 51,535 sequences per sample; 1,578,796 ITS sequences were obtained, with an average of 65,783 samples per sample. Root, shoot, leaf, and rhizosphere soil samples of CK wheat contained an average of 45,815, 44,742.7, 44,682, and 62,769 effective 16S rDNA sequences, including 67,791.33, 62,906, 67,970, and 71,551.67 effective ITS sequences. Roots shoot, leaf, and rhizosphere soil samples of wheat inoculated with JC-K3 contained, on average, effective 16S rDNA sequences of 41,945, 53,898.3, 58,866, and 60,563, including effective ITS sequences 66,696.33, 63,174, 54759.67, and 71416.33. As the number of sequencing increases, the microbial diversity index also gradually increases. At the final stage, the dilution curve became flat, indicating that the sequencing data for this study reached saturation and could cover most microbial communities in wheat roots, shoots, leaves, and rhizosphere soil (Supplementary Figure S2).

Diversity index analysis can be used to obtain information on species abundance, coverage, and diversity. Sobbing, Chaos, and Ace index reflect the richness of the community in the sample. As can be seen from Table 2, inoculation of the JC-K3 strain significantly increased the richness of bacterial community in shoots and leaves, and reduced the richness of fungal community in shoots and leaves compared with the control. At the same time, we found that inoculation with JC-K3 strain significantly increased the fungal community richness in wheat rhizosphere soil (*p* < 0.05).

**Table 2:** Diversity and richness indices of wheat root (R), shoot (Sh), leaf (L), and rhizospheric soil (S) samples under different treatments.

	Sample	sobs	chao	ace	Shannon	Simpson	Coverage
Bacteria	CK-R	82.33 ± 24.23	151.67 ± 49.24	119.91 ± 32.05	1.01 ± 0.04	0.6131 ± 0.0431	0.9992 ± 0.0004
	JC-K3-R	111.00 ± 29.53	135.76 ± 18.91	156.76 ± 24.25	0.90 ± 0.44	0.6948 ± 0.1606	0.9991 ± 0.0002
	CK-Sh	88.67 ± 26.04	114.90 ± 50.01	140.36 ± 80.51	0.72 ± 0.11	0.7459 ± 0.0397^*^	0.9991 ± 0.0005^*^
	JC-K3-Sh	1914.67 ± 18.45^*^	2150.55 ± 42.27^*^	2138.67 ± 29.60^*^	6.43 ± 0.03^*^	0.0040 ± 0.0003	0.9884 ± 0.0005
	CK-L	510.00 ± 210.64	703.76 ± 51.68	753.71 ± 229.59	2.91 ± 1.04	0.2259 ± 0.1730^*^	0.9942 ± 0.0022^*^
	JC-K3-L	1894.00 ± 31.82^*^	2155.72 ± 38.69^*^	2152.49 ± 25.59^*^	6.28 ± 0.12^*^	0.0058 ± 0.0015	0.9874 ± 0.0009
	CK-S	1862.00 ± 19.82	2120.60 ± 29.67	2114.01 ± 27.74	6.27 ± 0.05	0.0053 ± 0.0010	0.9877 ± 0.0004^*^
	JC-K3-S	1674.00 ± 132.05	2045.96 ± 100.53	2019.07 ± 82.05	5.82 ± 0.39	0.0103 ± 0.0041^*^	0.9861 ± 0.0007
Fungus	CK-R	142 ± 19.61	156.36 ± 25.01	151.05 ± 16.40	3.15 ± 0.84	0.1715 ± 0.1068	0.9997 ± 0.0001
	JC-K3-R	60 ± 59.42	64.33 ± 63.41	64.43 ± 63.09	1.50 ± 1.03	0.4783 ± 0.2760	0.9998 ± 0.0002
	CK-Sh	178.33 ± 30.14^*^	180.90 ± 31.29	181.49 ± 30.69	3.58 ± 0.81^*^	0.0969 ± 0.0880	0.9999 ± 0.0000^*^
	JC-K3-Sh	97.33 ± 5.19	125.25 ± 12.82	132.18 ± 3.24	1.41 ± 0.54	0.4144 ± 0.1868	0.9993 ± 0.0001
	CK-L	93.33 ± 44.53	98.90 ± 40.43	103.37 ± 35.15	1.59 ± 0.85	0.4732 ± 0.2705	0.9998 ± 0.0001^*^
	JC-K3-L	92.00 ± 17.72	124.13 ± 30.25	145.65 ± 26.64	0.66 ± 0.31	0.7873 ± 0.1108	0.9994 ± 0.0001
	CK-S	26.33 ± 10.78	27.40 ± 11.20	30.15 ± 8.55	0.62 ± 0.65	0.7433 ± 0.2917	0.9999 ± 0.0000^*^
	JC-K3-S	127.67 ± 41.61^*^	191.21 ± 59.55^*^	205.42 ± 61.72^*^	1.39 ± 0.34	0.5789 ± 0.1187	0.9993 ± 0.0002

Values are means ± SD.

* In the table represent the significant difference between the indexes of uninoculated and inoculated JC-K3 wheat, *p* < 0.05.

The Shannon index (the larger the value, the higher the community diversity) and the Simpson Index (the larger the value, the lower the community diversity) index reflect the community diversity in the sample. As can be seen from Table 2, inoculation with JC-K3 strain significantly increased the diversity of bacterial community in shoots and leaves, and reduced the diversity of fungal community in shoots and leaves (*p* < 0.05) compared with the control. The results showed that the richness and diversity of bacterial and fungal communities in wheat roots decreased after inoculation with JC-K3 strain, but the difference was not significant (Table 2).

### Comparison of Bacterial and Fungal Communities in JC-K3 and Controls

Venn diagrams can be used to visualize the similarity and overlap between species (such as OTUs) in environmental samples. In order to obtain the species classification information corresponding to each OTU, the RDP classifier Bayes algorithm was used to classify and analyze 97% similar OTU representative sequences. After inoculation with JC-K3, the unique and shared OTUs in wheat roots, shoot, leaves, and rhizosphere soils changed significantly (Supplementary Figure S3), while the unique and shared OTUs between the same tissues of wheat and rhizosphere soil was also observed under different treatments (Supplementary Figure S4).

**Figure 4:**
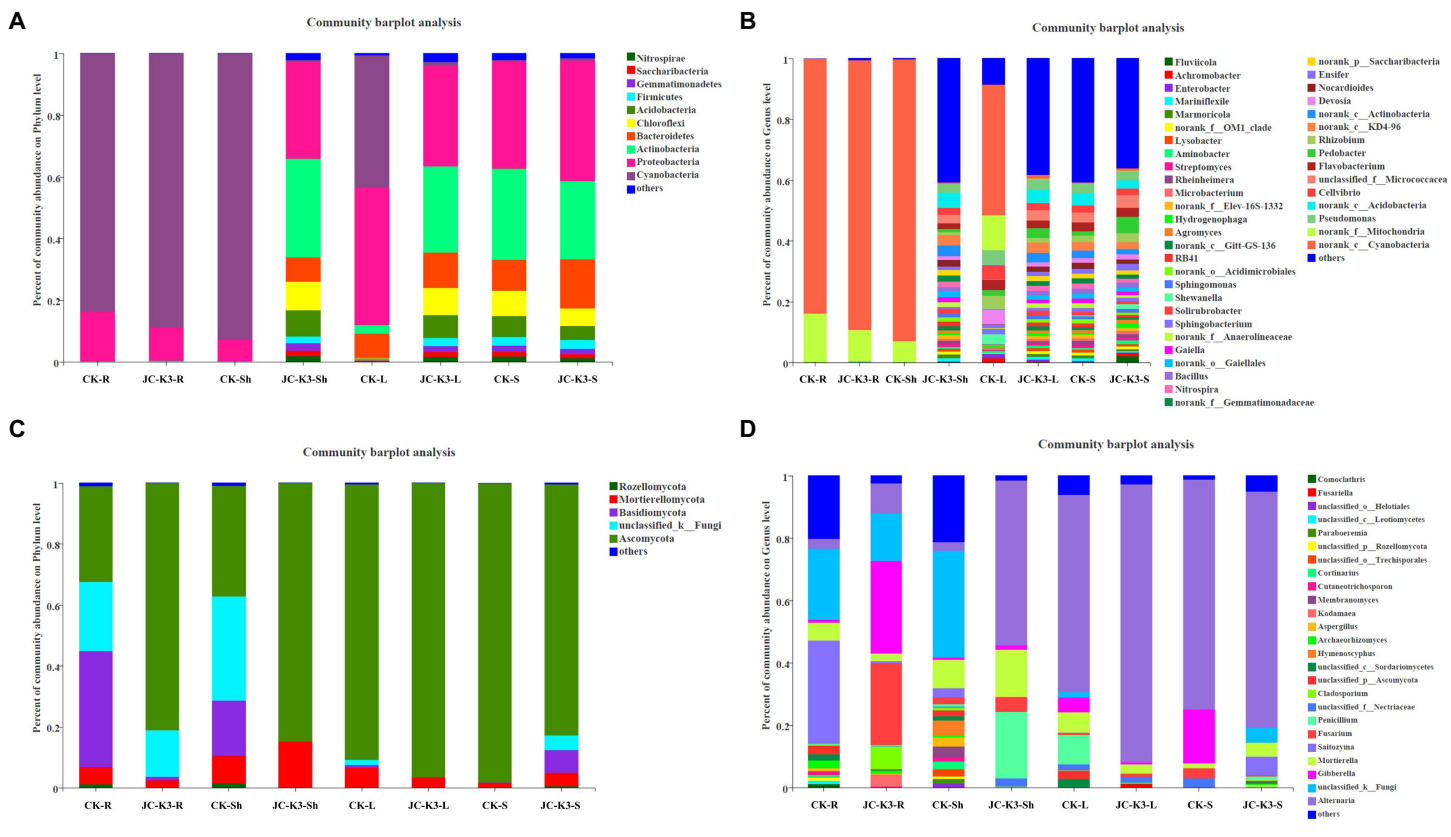
Community structure of bacterial and fungal in two groups. The stacked bar graphs represent the relative abundances of the major phyla and genera. **(A)** Differences in bacterial community structure at the phylum level with different treatments; **(B)** Differences in bacterial community structure at the genus level with different treatments; **(C)** Differences in fungal community structure at the phylum level under different treatments; and **(D)** Differences in fungal community structure at the genus level with different treatments. CK and JC-K3 represent the control and JC-K3 inoculated plants, respectively. S, soil; R, roots; Sh, shoots; and L, leaves.

**Figure 5:**
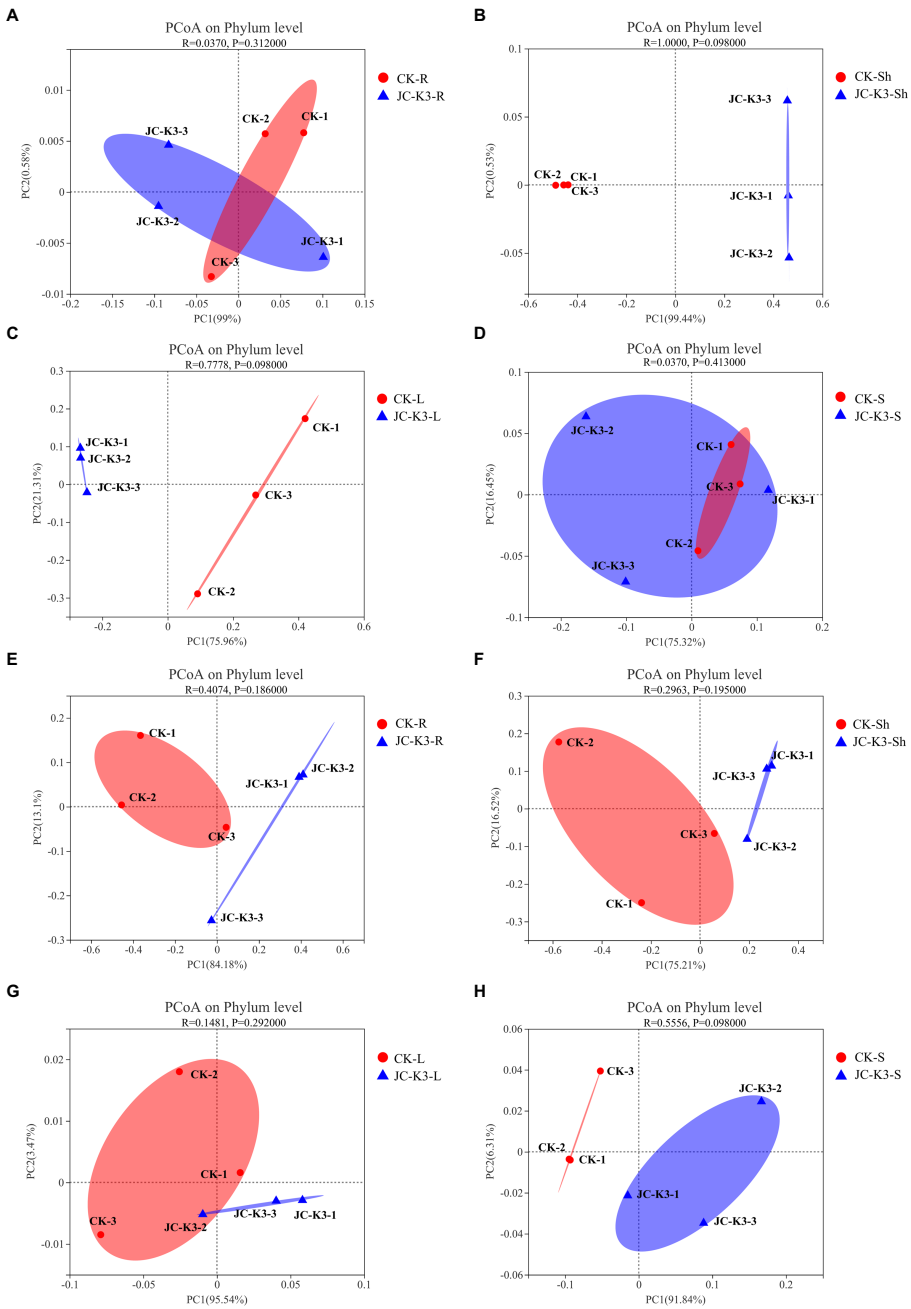
ContinuedFIGURE 5The principal co-ordinates analysis (PCoA) analysis based on Bray-Curtis distance was used to study the community composition of bacteria and fungi in plant roots, shoots, leaves, and rhizosphere soils inoculated with JC-K3 strain. **(A)** PCoA analysis of endophytic bacterial communities in wheat roots at phylum level; **(B)** PCoA analysis of endophytic bacterial community in wheat stem at phylum level; **(C)** PCoA analysis of endophytic bacterial community in wheat leaves at phylum level; **(D)** PCoA analysis of bacterial community in wheat rhizosphere at phylum level; **(E)** PCoA analysis of endophytic fungal communities in wheat roots at phylum level; **(F)** PCoA analysis of endophytic fungal communities in wheat stems at phylum level; **(G)** PCoA analysis of endophytic fungal communities in wheat leaves at phylum level; and **(H)** PCoA analysis of fungal communities in wheat rhizosphere at phylum level. CK and JC-K3 represent the control and JC-K3 inoculated plants, respectively. S, soil; R, roots; Sh, shoots; and L, leaves.

The Mothur program was used to classify all bacterial sequences by genus level and compare the relative abundances of the assigned phyla and genera between the samples (Figure 4). With regard to the community information of endophytic bacteria in the roots of at the phylum level, the relative abundance of *Cyanobacteria* in wheat roots inoculated with JC-K3 (JC-K3-R) accounting for 88.79%, but only 83.69% in the CK. while the relative abundance of *Proteobacteria* in the JC-K3-R group was 10.81%, which was lower than that observed in the CK (16.15%; Supplementary Figures S5A,B). Similarly, with regard to the community of endophytic bacteria in wheat shoots at the phylum level, the relative abundance of *Cyanobacteria* in wheat shoots inoculated with JC-K3 decreased; while the relative abundances of *Proteobacteria*, *Actinobacteria*, and *Bacteroidetes* increased when compared with CK (Supplementary Figures S5C,D). Supplementary Figures S5E,F show information on the community of endophytic bacteria in wheat leaves at the phylum level. The relative abundance of endophytic bacteria in wheat leaves changed significantly before and after inoculation with JC-K3 strain. Compared with CK, the relative abundance of *Proteobacteria* and *Cyanobacteria* in wheat leaves inoculated with JC-K3 decreased, while the relative abundance of *Bacteroidetes* and *Actinobacteria* increased. Studies on the information of the rhizosphere bacteria community in wheat rhizosphere soil at the phylum level show that after inoculation with JC-K3, the relative abundance of *Proteobacteria*, *Cyanobacteria*, and *Bacteroidetes* in wheat rhizosphere soil increased; the relative abundance of *Actinobacteria i* decreased (Supplementary Figures S5G,H). Of the classifiable fungal sequences, compared with CK, the relative abundance of *Ascomycota* in wheat roots inoculated with JC-K3 increased, while the relative abundance of *Basidiomycota*, *Mortierellomycota* decreased (Supplementary Figures S6A,B). Compared with CK, the relative abundance of *Mortierellomycota* and *Ascomycota* in wheat shoots inoculated with JC-K3 increased, while the relative abundance of *Rozellomycota* and *Basidiomycota* decreased (Supplementary Figures S6C,D). Compared with CK, the relative abundance of *Ascomycota* in wheat leaves inoculated with JC-K3 increased, while the relative abundance of *Mortierellomycota* and *Basidiomycota* decreased (Supplementary Figures S6E,F). Compared with CK, the relative abundance of *Rozellomycota*, *Mortierellomycota*, and *Basidiomycota* in wheat rhizosphere soil inoculated with JC-K3 increased, while the relative abundance of *Ascomycota* decreased (Supplementary Figures S6G,H). Based on the phylum level results of PCoA, there was a significant separation of fungal communities in wheat rhizosphere soil and roots under JC-K3 and CK treatments, while there was no significant difference in bacterial communities (Figures 5A,D,E,H). There was a significant separation of bacterial communities in JC-K3-L and CK-L, while there was no significant difference in fungal communities (Figures 5C,G). PCoA also indicated differences of bacterial and fungal communities between JC-K3-Sh and CK-Sh (Figures 5B,F), and ANOSIM analysis further confirmed the significant structural reorganization (Table 3). The PCOA results at genus level were similar to those at phylum level, except that JC-K3-R and CK-R were not significantly separated at genus level (Supplementary Figures S7A,E).

**Table 3:** Analysis of similarity (ANOSIM).

Phylogenetic level ANOSIM output		Phylum		OTU	
		Statistic	*p* value	Statistic	*p* value
*Bacillus*	Ctrl-rhizosphere soil vs. JC-K3-rhizosphere soil	0.037	0.413	0.2222	0.209
	Ctrl-rhizosphere soil vs. Ctrl-root	1.0000	0.098	1.0000	0.098
	Ctrl-rhizosphere soil vs. Ctrl-shoot	1.0000	0.098	1.0000	0.098
	Ctrl-rhizosphere soil vs. Ctrl-leaf	0.7778	0.098	0.7778	0.098
	Ctrl-root vs. JC-K3-root	0.037	0.312	0.1852	0.312
	Ctrl-root vs. Ctrl-shoot	0.4444	0.098	0.5185	0.098
	Ctrl-root vs. Ctrl-leaf	0.5185	0.098	0.5185	0.098
	Ctrl-shoot vs. JC-K3 shoot	1.0000	0.098	1.0000	0.098
	Ctrl-shoot vs. Ctrl-leaf	0.5556	0.098	0.5556	0.098
	Ctrl-leaf vs. JC-K3-leaf	0.7778	0.098	0.7778	0.098
	JC-K3-rhizosphere soil vs. JC-K3-root	1.0000	0.098	1.0000	0.098
	JC-K3-rhizosphere soil vs. JC-K3-shoot	0.1481	0.413	0.4074	0.098
	JC-K3-rhizosphere soil vs. JC-K3-leaf	0.1111	0.406	0.2222	0.208
	JC-K3-root vs. JC-K3-shoot	1.0000	0.098	1.0000	0.098
	JC-K3-root vs. JC-K3-leaf	1.0000	0.098	1.0000	0.098
	JC-K3-shoot vs. JC-K3-leaf	0.2593	0.297	0.4444	0.098
Fungus	Ctrl-rhizosphere soil vs. JC-K3-rhizosphere soil	0.5556	0.098	0.1852	0.201
	Ctrl-rhizosphere soil vs. Ctrl-root	0.7778	0.098	0.8519	0.098
	Ctrl-rhizosphere soil vs. Ctrl-shoot	0.5556	0.098	0.963	0.098
	Ctrl-rhizosphere soil vs. Ctrl-leaf	0.2593	0.289	0.1111	0.201
	Ctrl-root vs. JC-K3-root	0.4094	0.186	0.5185	0.098
	Ctrl-root vs. Ctrl-shoot	−0.1481	0.697	0.3333	0.298
	Ctrl-root vs. Ctrl-leaf	0.7778	0.098	0.8148	0.098
	Ctrl-shoot vs. JC-K3-shoot	0.2963	0.195	0.963	0.098
	Ctrl-shoot vs. Ctrl-leaf	0.5185	0.098	1.0000	0.098
	Ctrl-leaf vs. JC-K3-leaf	0.1481	0.292	0.2963	0.195
	JC-K3-rhizosphere soil vs. JC-K3-root	0.037	0.289	0.5556	0.098
	JC-K3-rhizosphere soil vs. JC-K3-shoot	0.1852	0.209	0.4815	0.098
	JC-K3-rhizosphere soil vs. JC-K3-leaf	0.5556	0.098	0.4815	0.098
	JC-K3-root vs. JC-K3-shoot	−0.037	0.575	0.3704	0.098
	JC-K3-root vs. JC-K3-leaf	−0.1852	1.000	0.3333	0.098
	JC-K3-shoot vs. JC-K3-leaf	−0.037	0.598	0.5556	0.098

The overall compositions of bacteria in wheat roots and rhizosphere soil were similar at the genus level, whereas the distribution in wheat shoot and leaf varied (Figure 4B). Of the classifiable bacterial sequences, almost no dominant bacterial genera were found in CK-Sh group, while *Nitrospira* (1.87%), *Pseudomonas* (2.92%), and other dominant bacterial genera were found in JC-K3-Sh group. The distribution of *Flavobacterium*, *Rhizobium*, *Cellvibrio*, and *Pseudomonas* accounting for 3.46, 4.45, 4.59, and 4.85% in the CK-L group, but only 2.44, 1.52, 2.34, and 3.44% in the JC-K3-L group, respectively (Figure 4B). Of the classifiable fungal sequences, the proportion of *Penicillium* was 21.34% in the JC-K3-Sh group, which was higher than that observed in the CK-Sh (0.00%) group. The proportion of *Fusarium* and *Gibberella* in the JC-K3-S group was 0.35 and 0.05%, which was lower than that in the CK-S (3.24 and 17.25%; Figure 4D).

**Figure 6:**
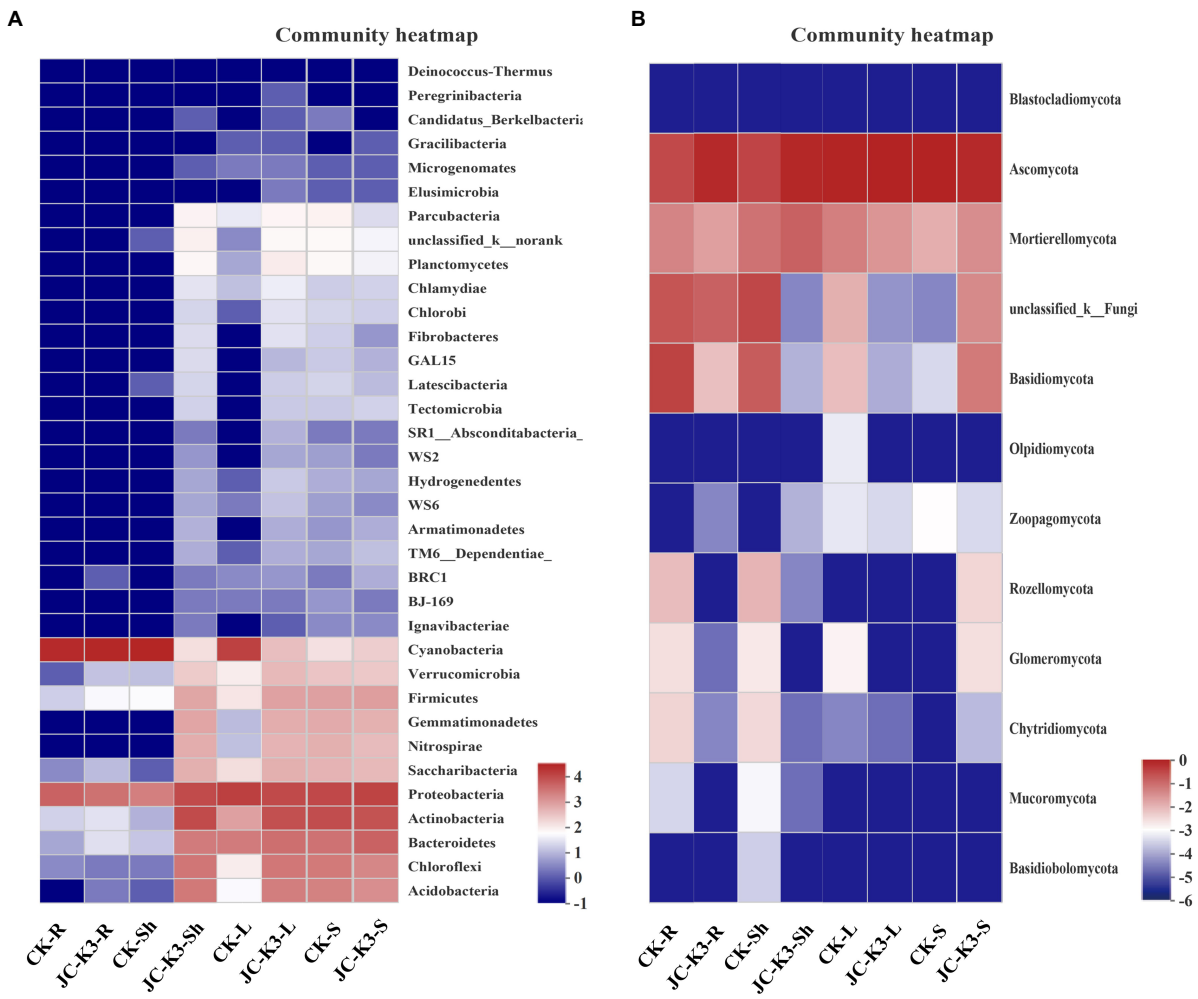
Hierarchically clustered heatmaps of the bacterial **(A)** and fungal **(B)** distributions. The heatmaps show the distribution of different communities from two groups at the phylum level. CK and JC-K3 represent the control and JC-K3 inoculated plants, respectively. S, soil; R, roots; Sh, shoots; and L, leaves.

Based on the heat map of the phylum level species and cluster tree analysis of the samples, the differences in composition of bacterial and fungal community in the roots, shoots, leaves, and rhizosphere soil of plants inoculated with and without JC-K3 strain were studied (Figure 6). The results showed that the bacterial community composition in JC-K3-Sh, JC-K3-S, JC-K3-L, and CK-S was similar, and there were differences with CK-R, JC-K3-R, and CK-Sh (Figure 6A). The proportions of *Rozellomycota*, *Glomeromycota*, *Chytridiomycota*, and *Mucoromycota* in JC-K3-R and JC-K3-Sh were significantly lower than that in CK-R and CK-Sh (Figure 6B).

**Figure 7:**
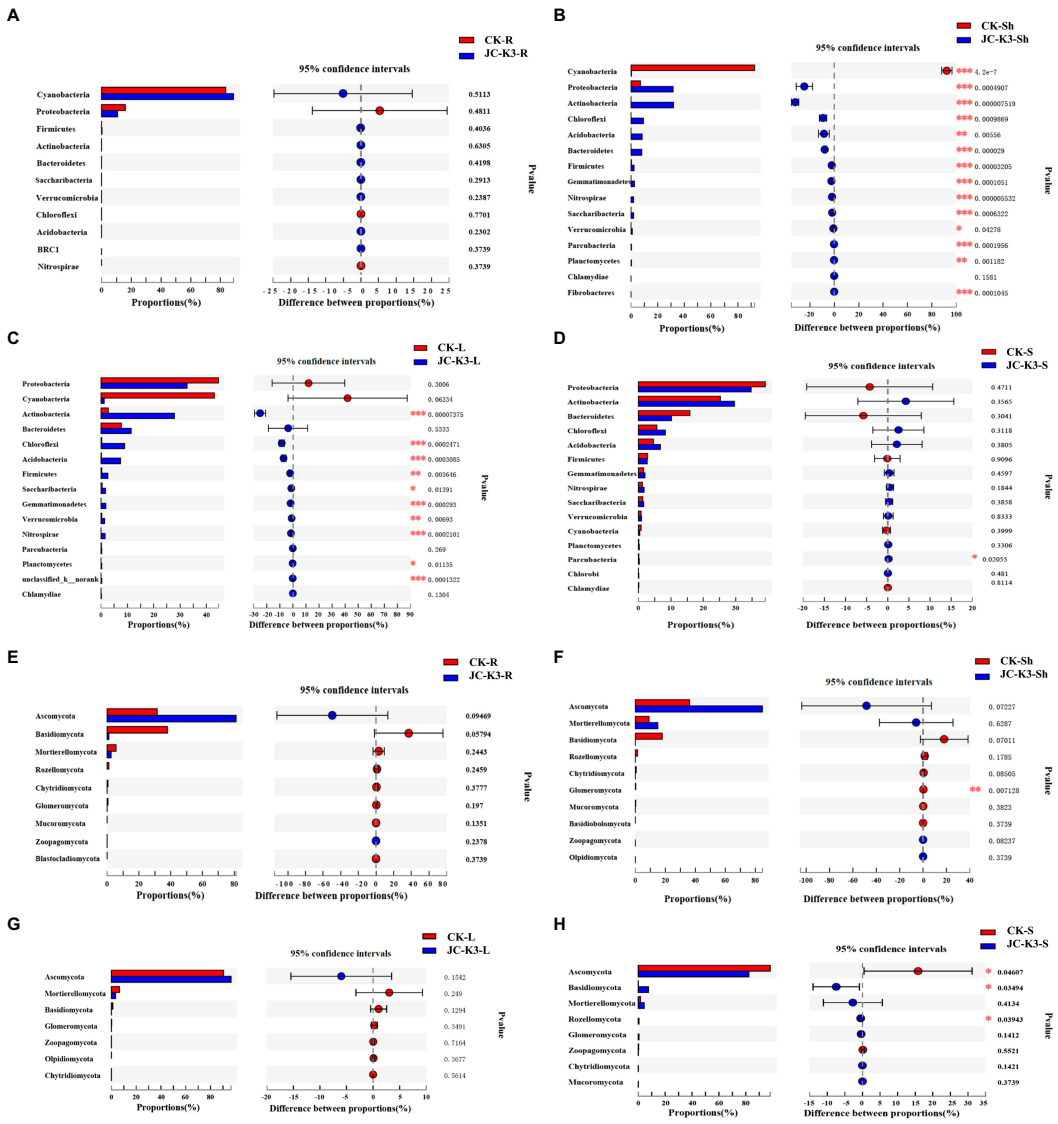
The significant differences in microbial groups at the phylum level among the root, shoot, leaf, and rhizosphere soils of wheat treated with different treatments (Student’s *t*-test bar plot on phylum level). Compared with the control group, **(A)** the difference of endophytic bacteria in wheat roots at phylum level; **(B)** the difference of endophytic bacteria in wheat shoots at phylum level; **(C)** the difference of endophytic bacteria in wheat leaves at phylum level; **(D)** the difference of rhizosphere bacteria in wheat rhizosphere soil at phylum level; **(E)** the difference of endophytic fungi in wheat roots at phylum level; **(F)** the difference of endophytic fungi in wheat shoots at phylum level; **(G)** the difference of endophytic fungi in wheat leaves at phylum level; and **(H)** the difference of rhizosphere fungi in wheat rhizosphere soil at phylum level. CK and JC-K3 represent the control and JC-K3 inoculated plants, respectively. S, soil; R, roots; Sh, shoots; and L, leaves. The rightmost is *p* value, ^*^0.01 < *p* ≤ 0.05, ^**^0.001 < *p* ≤ 0.01, and ^***^p ≤ 0.001.

Through the analysis of interspecies differences, it was found that at the phylum level, there were significant differences between endophytes and rhizosphere microorganisms in wheat inoculated with CK and JC-K3. As shown in Figure 7, the Student’s *t*-test was used to test for significant differences in bacteria and fungi in the same tissue of endophytic bacteria or wheat treated with different treatment methods. There was no significant difference in the content of endophytic bacteria in wheat roots after JC-K3 inoculation when compared with the control (Figure 7A). The contents of *Cyanobacteria* in the shoots of wheat were significantly decreased after inoculation with JC-K3 (*p* ≤ 0.001), while the contents of *Proteobacteria*, *Actinobacteria*, *Chloroflexi*, *Bacteroidetes*, *Firmicutes*, *Gemmatimonadetes*, *Nitrospirae*, *Saccharibacteria*, and *Parcubacteria* were significantly increased (*p* ≤ 0.001; Figure 7B). The contents of *Actinobacteria*, *Chloroflexi*, *Acidobacteria*, *Gemmatimonadetes*, and *Nitrospirae* in wheat leaves were significantly increased after inoculation with JC-K3 (*p* ≤ 0.001; Figure 7C). The content of *Parcubacteria* in rhizosphere soil decreased significantly (*p* ≤ 0.05; Figure 7D). After inoculation with JC-K3, there was no significant difference in the content of endophytic fungi in wheat roots and leaves (Figures 7E,G). The content of *Glomeromycota* in wheat shoots decreased significantly (*p* ≤ 0.05; Figure 7F); the content of *Ascomycota* in rhizosphere soil of wheat decreased significantly, and the content of *Basidiomycota* and *Rozellomycota* increased significantly (*p* ≤ 0.05; Figure 7H).

Through the analysis of interspecies differences at genus level, there was no significant difference in the content of endophytic bacteria in wheat roots and rhizosphere soil after JC-K3 inoculation when compared with the control (Supplementary Figures S8A,D). The contents of *Nitrospira*, *Gaiella*, *Bacillus*, *Sphingomonas*, and *Lysobacter* were significantly increased in the shoots and leaves of wheat after inoculation with JC-K3 (*p* ≤ 0.001; Supplementary Figures S8B,C), but there was no significant difference in the content of endophytic fungi (Supplementary Figures S8E,G). The content of *Penicillium* in wheat shoots significantly increased (*p* ≤ 0.001; Supplementary Figure S8F); the content of *Penicillium* and *Cladosporium* in rhizosphere soil of wheat increased significantly (*p* ≤ 0.001; Supplementary Figure S8H).

## Discussion

*Bacillus velezensis* originates from the rhizosphere, soil, plant interior, rivers, and other ecological environments of plants. Most *Bacillus velezensis* isolated from the plant rhizosphere can colonize the roots of plant and play an important role in suppressing pathogenic bacteria (Wu et al., 2015). In recent years, several *Bacillus velezensis* strains have been isolated at home and abroad, and their roles in plant growth, disease and insect resistance, and induction system resistance have been studied. It is believed that many *Bacillus velezensis* are thought to play an important role in biological control and increasing food production. Interestingly, it is known that *Bacillus velezensis* has high salt tolerance, but its effect on saline-alkaline soils is little known. *Bacillus velezensis* produces substances such as IAA and NH_3_, which can promote the growth of beets, carrots, cucumbers, peppers, potatoes, turnips, pumpkins, tomatoes, and radishes, and inhibit *Streptomyces scabies* (Meng et al., 2016). The present study demonstrates the effectiveness of *Bacillus velezensis* JC-K3 for improving growth of wheat plants under salt stress conditions.

In this study, we identified the PGP activity of the isolated JC-K3 and found that the strain had significant IAA, ACC deaminase, siderophore, and glucanase production capacities (Table 1). These are considered to be PGP traits because they have the ability to provide P to plants under P-limiting conditions, promote plant growth by acting as a phytohormone (IAA), provide Fe to plants through chelation and absorption (siderophores), and deplete precursor to the plant stress hormone ethylene (ACC deaminase). The results showed that JC-K3 can play a role in saline-alkali soil, effectively improve wheat biomass accumulation and osmotic regulation ability, and significantly increase Na and K absorption in wheat. Thus, our report extends the understanding of plant growth promoting properties contributed by members of genus *Bacillus*.

Plants in saline-alkali land have a certain ability to repair. In particular, they have also developed the ability to take advantage of the benefits provided by endophytes and rhizosphere microorganisms (Trivedi et al., 2020). Strains isolated from saline-alkali environments have significant effects on plant growth under salt stress (Ullah and Bano, 2015). A significant decrease in shoot/root lengths and fresh/dry weight was observed in uninoculated plants under salt stress, whereas inoculation with JC-K3 limited these losses significantly. It is likely that this response might be due to ACCD activity of the bacterium. Our results are in agreement with the previous report for salt tolerance in various plants induced by PGPR (Singh and Jha, 2016). Compared with uninoculated plants, after inoculation in salt-tolerant PGPB, these plants have increased accumulation of osmotic substances (such as sugar) and antioxidant enzyme activities (such as SOD, peroxidase, CAT, and ascorbic acid peroxidase) increased. Similarly, Siddikee et al. (2010) showed that after inoculation with salt-tolerant PGPB, the growth of rape seedlings increased significantly, such as a 35–43% increase in dry weight and a 29–47% increase in length. We confirmed that the JC-K3 strain has a similar effect. PGPB can directly reduce the accumulation of toxic ions such as Na and Cl by regulating the expression and/or activity of ion transporters and improve the nutritional status of macronutrients and micronutrients. These results are in agreement with by earlier studies, which suggest that certain PGPR not only delay the uptake of Na^+^ but also improve the efficiency of inoculation of selected ions by inoculated plants to maintain a high K^+^/Na^+^ ratio (Etesami, 2018).

In many studies, the high homology between the 16S rRNA genes of bacteria, the 16S rRNA gene of chloroplasts, the 16S rRNA genes of plant nuclei and mitochondria, and the high abundance of 16S rRNA genes of chloroplast led to non-target unexpected co-amplification of the sequence. We use optimized PCR methods to reduce co-amplification of chloroplast and mitochondrial 16S rRNA to ensure that the 16S rRNA sequence of bacteria is correctly detected (Gottel et al., 2011; Bulgarelli et al., 2012). We observed differences in the dilution curves of rhizosphere soil and endosphere samples (Supplementary Figure S2). This may be due to sporadic and uneven colonization differences in wheat roots, shoots, leaves, and rhizosphere soil (Trivedi et al., 2020). Especially in rhizosphere soil, because root exudates and mucilage-derived nutrients attract countless organisms into the rhizosphere environment, plant-associated bacteria must have to be high competitiveness to successfully colonize in the root zone (Kong et al., 2020).

At the phylum level, *Actinomycetes* and *Proteobacteria* (mainly *Alpha*- and *Beta*-*proteobacteria*) are followed by *Bacteroidetes*, *Firmicutes*, and *Acidobacteria*. The ratio of *Proteobacteria* to *Acidobacteria* in rhizosphere communities has shown an indicator of soil nutrient content, in which *Proteobacteria* are associated with nutrient-rich soils and *Acidobacteria* are associated with nutrient-poor soils (Beckers et al., 2017). Similar to *Arabidopsis* (Bulgarelli et al., 2012), rice (Edwards et al., 2015), and poplar (Gottel et al., 2011), the relative abundance of *Acidobacteria* and *Actinobacteria* decreases from rhizosphere soil to rhizosphere microbiota. The relative abundance of *Proteobacteria* increased. Meanwhile, *Proteobacteria* include many bacteria responsible for nitrogen fixation (Wang et al., 2015). The relative abundance of *Proteobacteria* in rhizosphere soil of wheat inoculated with JC-K3 was higher than that of CK (Supplementary Figures S5G,H), indicating that this strain can increase the nutrient content of rhizosphere soil. Of the classifiable bacterial sequences, there was no significant difference in the relative abundance of dominant bacterial genera in rhizosphere communities after JC-K3 inoculation when compared with the control. However, the relative abundance of fungi genera, such as *Penicillium*, *Cladosporium*, and *Podospora* in rhizosphere soil was significantly increased. At the same time, the relative abundance of *Penicillium* in shoots of wheat inoculated with JC-K3 was significantly increased. Of these genera *Penicillium* have been used as biocontrol agents because of their numerous secondary metabolites (Larena et al., 2018).

In wheat shoots and leaves, inoculation of JC-K3 resulted in a significant increase of *Nocardioides*, *Nitrospira*, *Gaiella*, *Solirubrobacter*, *RB41*, *Bacillus*, *Sphingomonas*, *Marmoricola*, *Streptomyces*, *Mariniflexile*, and *Lysobacter*. Of these bacterial genera, the N-fixing functional trait of *Sphingomonas* spp. is of particular interest, suggesting their possible role in the promotion of plant growth (Agnolucci et al., 2019). Actually, the inoculation of wheat seeds with a strain of *Sphingomonas* sp. increased root biomass accumulation and the concentration of nutrients (Jia et al., 2018). At the same time, *Streptomyces* spp. showed promising PGP traits, being capable of solubilizing phytates and phosphates, and producing IAA and siderophores (Battini et al., 2016). Moreover, wheat plants grown in soil contaminated by the pathogenic fungus *Rhizoctonia solani*, inoculated with *Streptomyces* sp. F5, showed lower root damage and higher grain yield, compared to control group (Barnett et al., 2019). In particular, the relative abundance of beneficial microbes, such as *Pseudomonas* and *Rhizobium* were significantly increased in the CK-Sh group. It is important to note that many strains of different *Pseudomonas* species have long been known as PGP bacteria, playing also a key role as biocontrol agents (Schlatter et al., 2017). Moreover, *Pseudomonas* protected wheat plants against oxidative stress induced by ion, through the improvement of nutrients bioavailability, lowering of ion uptake, and elicitation of plant antioxidant responses (Islam et al., 2014). Overall, these results confirm that beneficial microbes accumulate more easily in the wheat shoots, leaves, and rhizosphere following application of JC-K3 compared with the control group.

Strains isolated from saline-alkali environments are more suitable for the soil environment of the inoculated area (Morgan, 2010; Debray et al., 2021). The JC-K3 strain isolated from the Yellow River delta was applied to the saline-alkali soil of wheat plantations in the Yellow River delta, and it can reflect the actual effect of the strain. However, there are still some shortcomings in this research, for example: (1) Although “jimai21”is a wheat variety widely promoted in this region, this study did not select other wheat varieties for comparative study; (2) Considering the limitations of wheat salt tolerance, the representative soil of the Yellow River delta was selected, but the soil with higher salt concentration and lower salt concentration was not selected for the experiment. (3) To verify the positive effect of JC-K3 inoculation on wheat growth, we compared the growth of wheat inoculated with JC-K3 and uninoculated with JC-K3, but did not test other isolated strains. This is mainly due to the significant differences, alkali resistance, and PGP activity between JC-K3 and other strains. Compared with previous studies, JC-K3 strain has the potential to improve plant salt tolerance and promote wheat growth, so this paper focuses on the role of this strain.

## Conclusion

The purpose of this study was to investigate the effect of JC-K3 strain isolated from the Yellow River Delta on wheat growth under salt stress. At the same time, the composition and diversity of endophytic bacteria and endophytic fungi in the roots, shoots, and leaves of wheat and the composition and diversity of rhizosphere bacteria and rhizosphere fungi were compared. JC-K3 strain improved wheat’s biomass accumulation ability, osmotic adjustment ability, and ion selective absorption ability. Not only that, JC-K3 significantly changed the diversity and abundance of endophytic and rhizosphere microorganisms in wheat. Although the conclusions reached in this study are limited due to the use of a single strain and plant host, the interaction between endophytic communities and plant salt tolerance needs further study. However, the results of this study provide a basis for understanding the response of wheat endophytic rhizosphere microorganisms to PGPB inoculation. At the same time, we believe that *Bacillus velezensis* can not only control plant diseases, but also can be used as a biological inoculant to reduce the plant salt stress damage.

## Data Availability Statement

The datasets presented in this study can be found in online repositories. The names of the repository/repositories and accession number(s) can be found at: NCBI – PRJNA642335, PRJNA643390.

## Author Contributions

CJ and XW conceived and designed the experiment. CJ, XS, QZ, CL, QG, and HL performed the experiment. CJ, JL, and PZ analyzed the data. CJ wrote the paper. ZC and HC guided the research work and thoroughly reviewed and corrected English language of the manuscript. All authors contributed to the article and approved the submitted version.

## Funding

This work was supported by the National Natural Science Foundation of China (32072518), Weifang University Doctor Initiation Fund Project (44121013), Major Science and Technology Innovation Project of Shandong province (2019JZZY020614), Shandong Agricultural Science and Technology Fund (Forestry, Science, and Technology Innovation; 2019LY003-5), West Coast Science and Technology Foundation of Qingdao (2019-23), Science and Technology Innovation and Development Special Project of Linyi city (2019ZDYF013), the National Natural Science Foundation of China (31770668), and Research and Reform Practice of New Agricultural Science of Ministry of Education (2-160).

## Conflict of Interest

The authors declare that the research was conducted in the absence of any commercial or financial relationships that could be construed as a potential conflict of interest.

## Publisher’s Note

All claims expressed in this article are solely those of the authors and do not necessarily represent those of their affiliated organizations, or those of the publisher, the editors and the reviewers. Any product that may be evaluated in this article, or claim that may be made by its manufacturer, is not guaranteed or endorsed by the publisher.

## Footnotes

^1^ http://rdp.cme.msu.edu/classifier/class_help.jsp#conf
