# Supplementary material for: Effect ofBacillus velezensis JC-K3 on Endophytic Bacterial and Fungal Diversity in Wheat Under Salt Stress
Source: Front Microbiol. 2021 Dec 20;12:802054. doi: 10.3389/fmicb.2021.802054 (PMC8722765; doi:10.3389/fmicb.2021.802054)
Supplement: Supplementary file 1 [file Data_Sheet_1.docx]

**Supplements:**


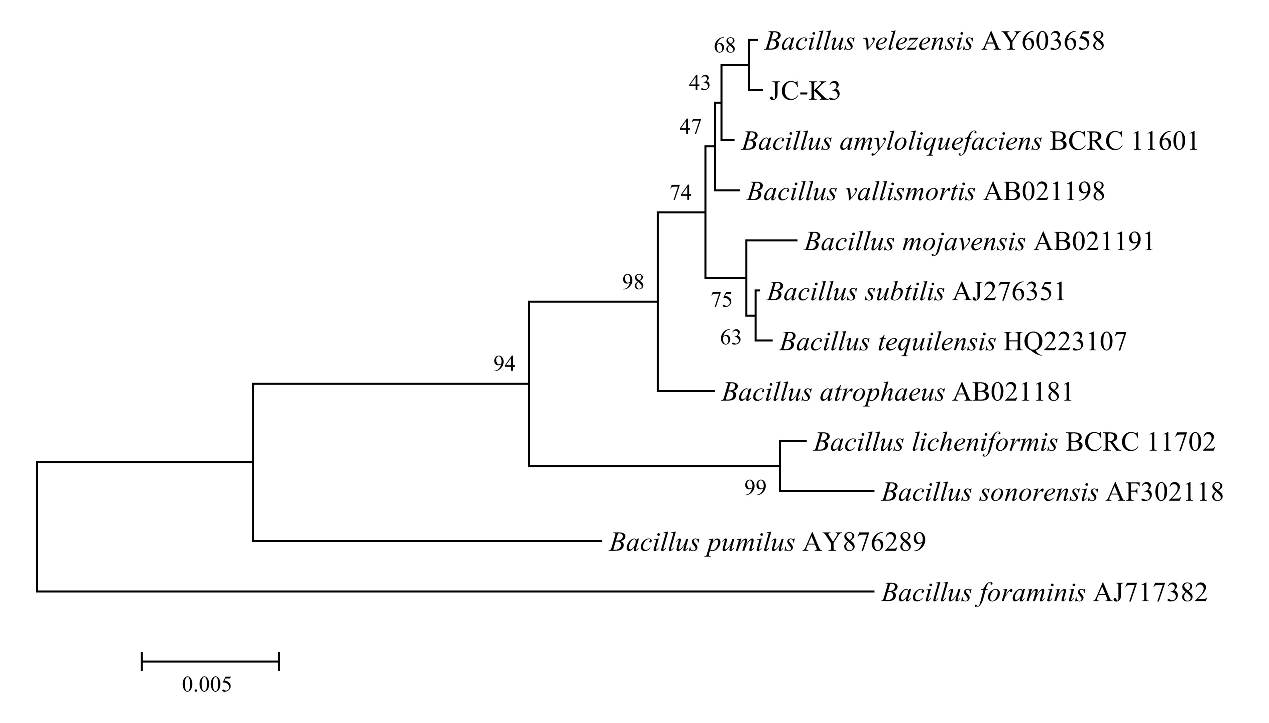


**Figure S1 | Phylogenetic tree shows relationship between JC-K3 and other strains. Bootstrap values indicated on nodes.**


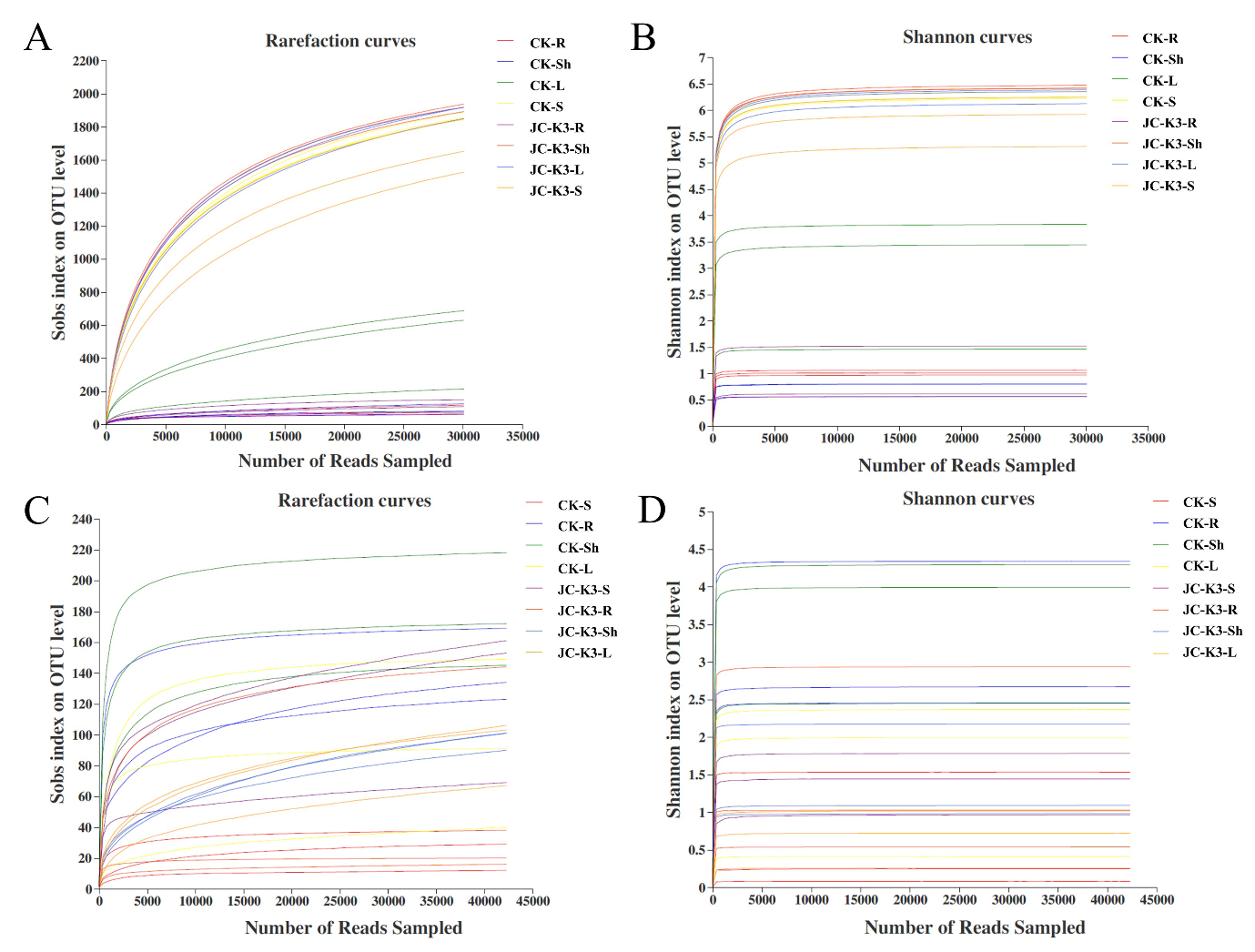


**Figure S2 | Different treatment samples generate microbial dilution curve and aroma curve based on OUT level.** (A) Rarefaction curves of bacteria from JC-K3 and control (CK) treatments; (B) Shannon curves of bacteria from JC-K3 and control treatments; (C) Rarefaction curves of fungi from JC-K3 and control treatments; (D) Shannon curves of fungi from JC-K3 and control treatments. The rarefaction curves depict the effects of a 3% dissimilarity cut-off on the number of OTUs identified in the two groups. CK and JC-K3 represent the control and JC-K3 inoculated plants, respectively. S, soil; R, roots; Sh, shoots; L, leaves.


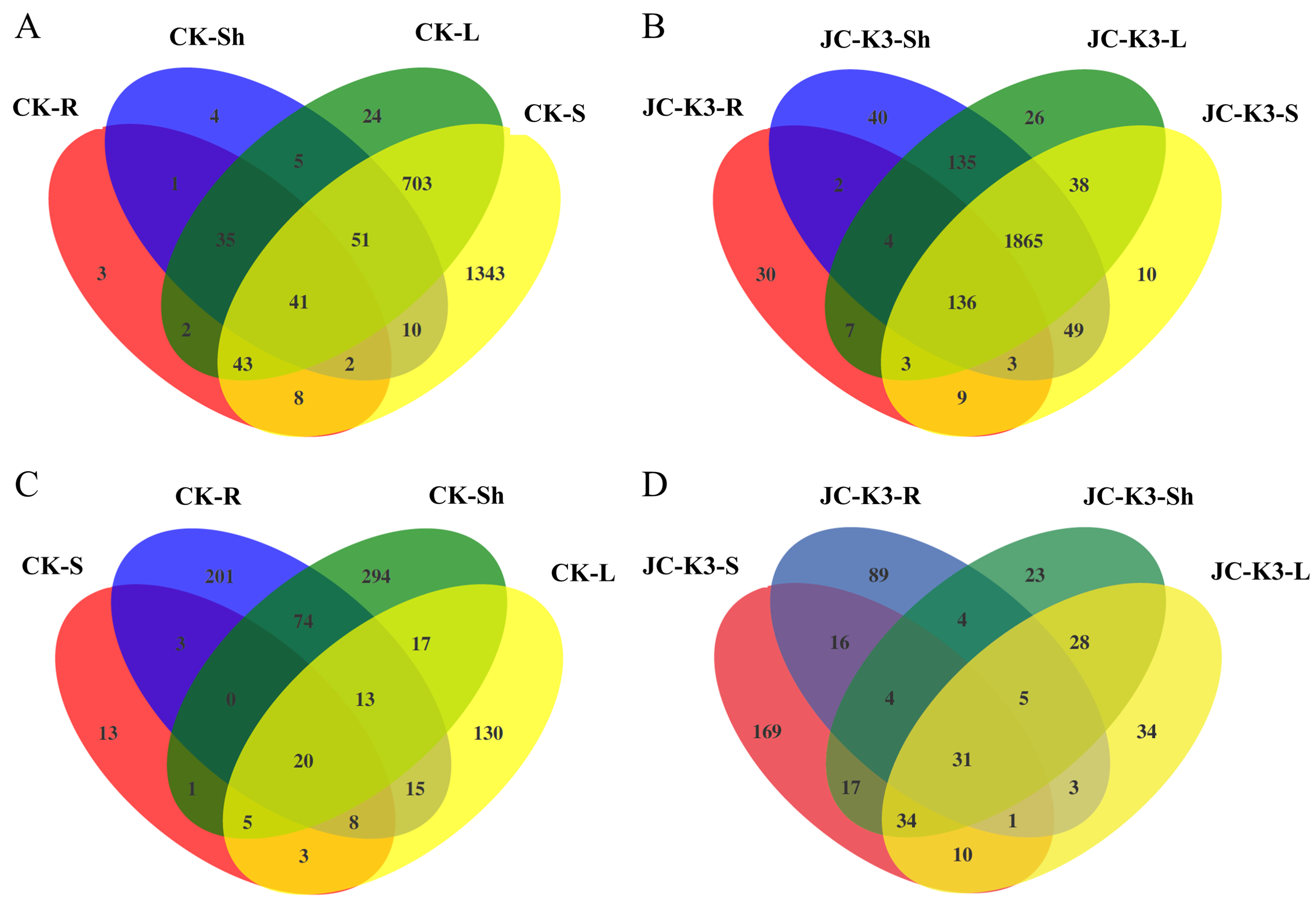


**Figure S3 | Venn diagram constructed at different OTUs levels.** (A) the number of bacterial OTUs unique and shared in the control group, (B) the number of bacterial OTUs unique and shared in the treatment group, (C) the number of fungal OTUs unique and shared in the control group, and (D) the number of fungal OTUs unique and shared in the control group. CK and JC-K3 represent the control and JC-K3 inoculated plants, respectively. S, soil; R, roots; Sh, shoots; L, leaves.


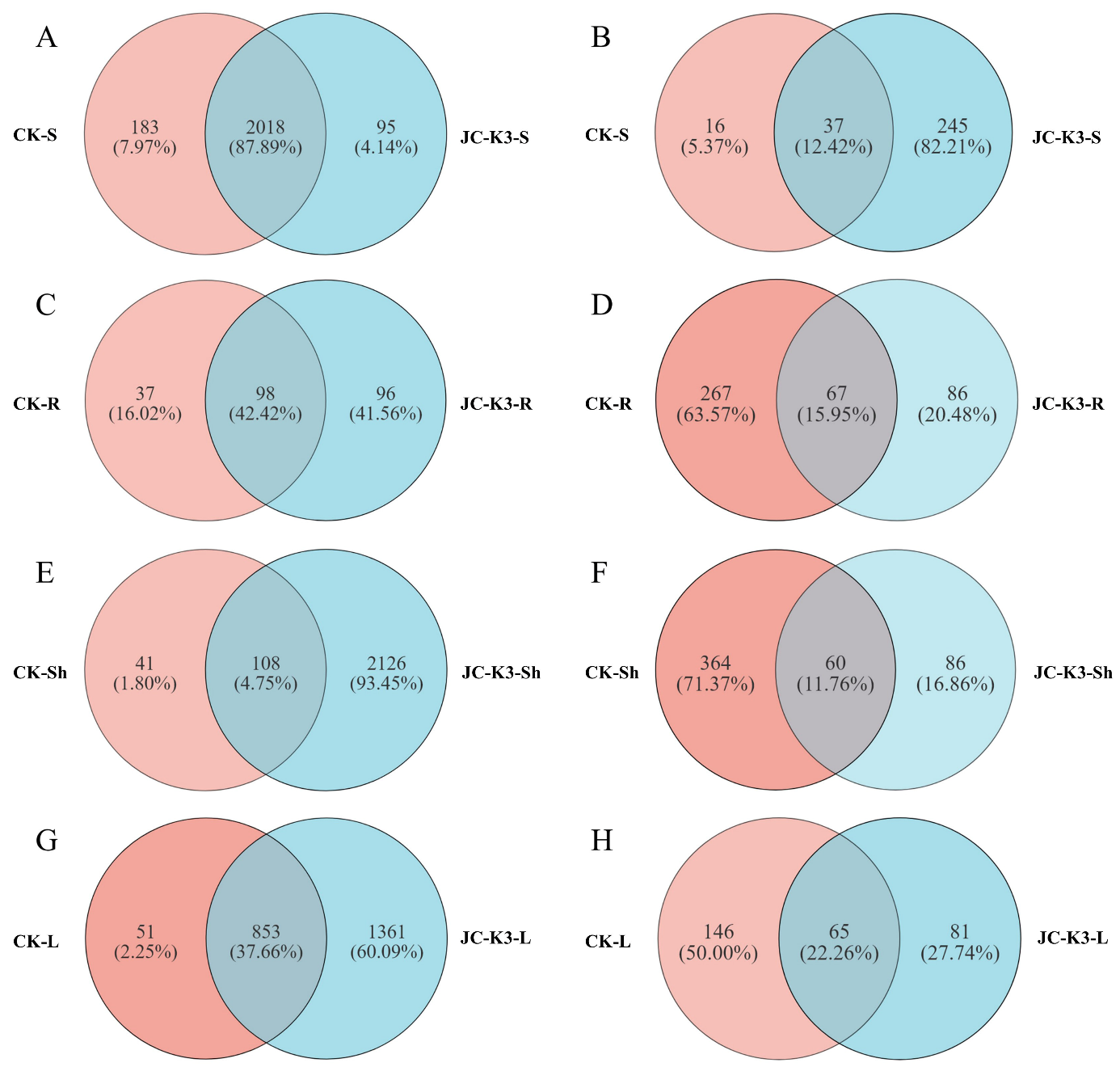


**Figure S4 | Venn diagram constructed the unique and shared OUTs between the same tissues of wheat and rhizosphere soil in different treatments.** (A) the number of unique and shared bacteria OTUs in rhizosphere soil in control and treatment groups; (B) number of root unique and shared bacteria OTUs in control and treatment groups; (C) number of shoot unique and shared bacteria OTUs in control group and treatment group; (D) the number of unique and shared bacteria OTUs in the control group and the treatment group; (E) number of unique and shared fungi OTUs in rhizosphere soil in control and treatment groups; (F) number of root unique and shared fungi OTUs in control and treatment groups; (G) number of shoot unique and shared fungi OTUs in control group and treatment group; (H) number of unique and shared fungi OTUs in the control group and treatment group. CK and JC-K3 represent the control and JC-K3 inoculated plants, respectively. S, soil; R, roots; Sh, shoots; L, leaves.


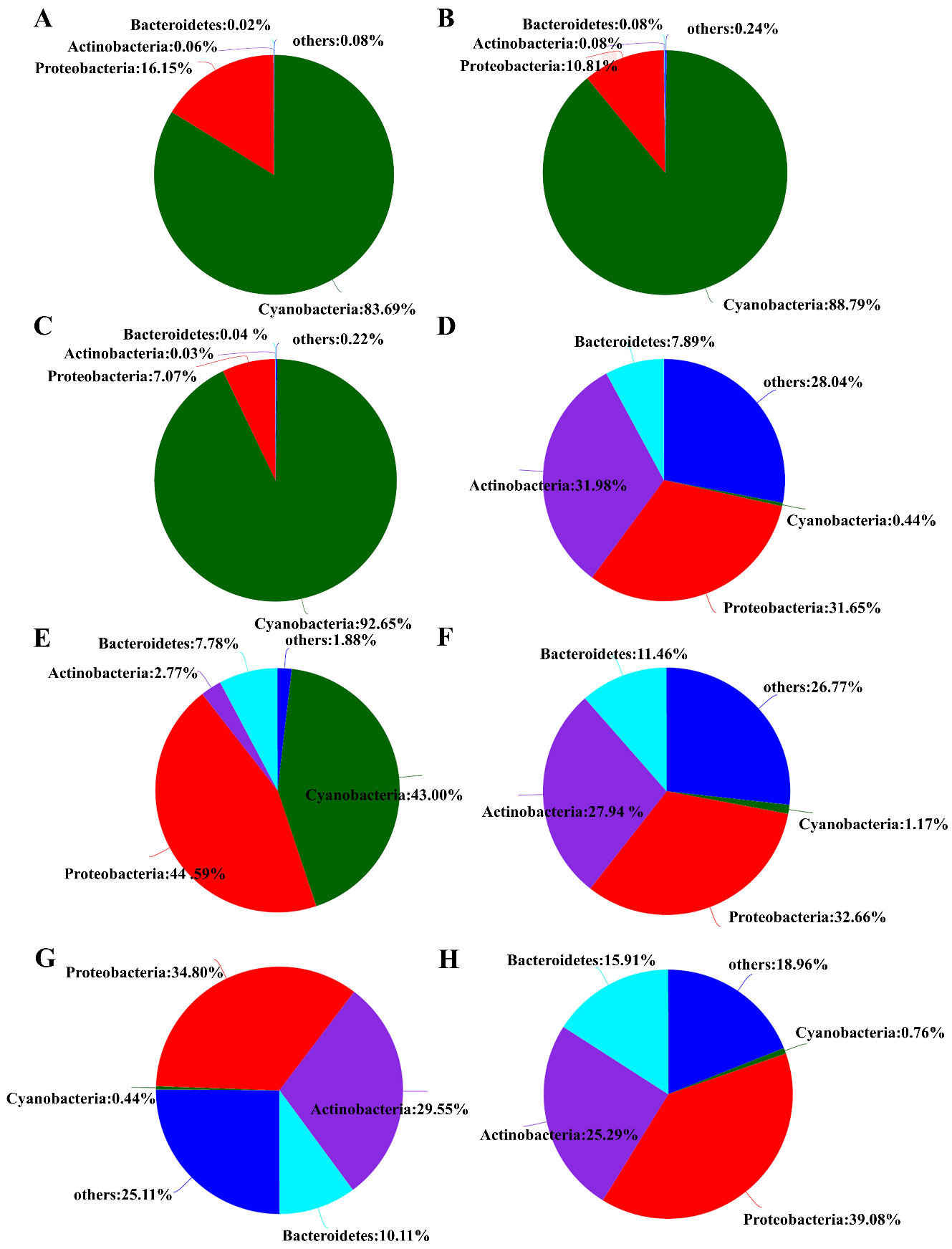


**Figure S5 | The relative abundance (%) of endogenous bacterial phyla in wheat.** (A) relative abundance of endophytic bacteria in wheat roots of control group; (B) relative abundance of endophytic bacteria in wheat roots inoculated with JC-K3 strain; (C) relative abundance of endophytic bacteria in wheat shoots of control group; (D) relative abundance of endophytic bacteria in wheat shoots inoculated with JC-K3 strain; (E) relative abundance of endophytic bacteria in wheat leaves of control group; (F) relative abundance of endophytic bacteria in wheat leaves inoculated with JC-K3 strain; (G) relative abundance of rhizobacteria in wheat rhizosphere soil of control group; (H) relative abundance of rhizobacteria in wheat rhizosphere soil inoculated with JC-K3 strain.


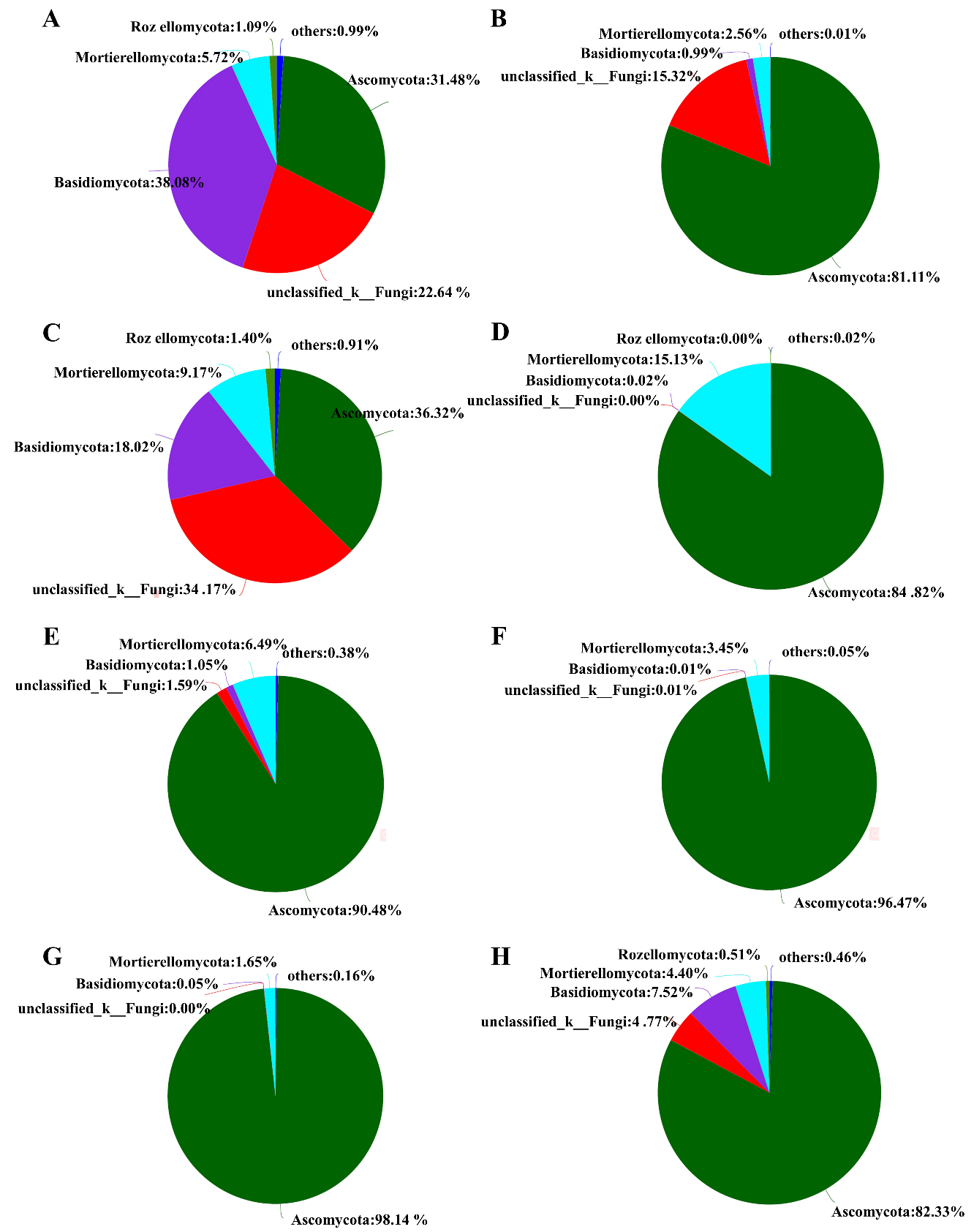


**Figure S6 | The relative abundance (%) of endogenous fungal phyla in wheat.** (A) relative abundance of endophytic fungal in wheat roots of control group; (B) relative abundance of endophytic fungal in wheat roots inoculated with JC-K3 strain; (C) relative abundance of endophytic fungal in wheat shoots of control group; (D) relative abundance of endophytic fungal in wheat shoots inoculated with JC-K3 strain; (E) relative abundance of endophytic fungal in wheat leaves of control group; (F) relative abundance of endophytic fungal in wheat leaves inoculated with JC-K3 strain; (G) relative abundance of rhizosphere fungi in wheat rhizosphere soil of control group; (H) relative abundance of rhizosphere fungi in wheat rhizosphere soil inoculated with JC-K3 strain.


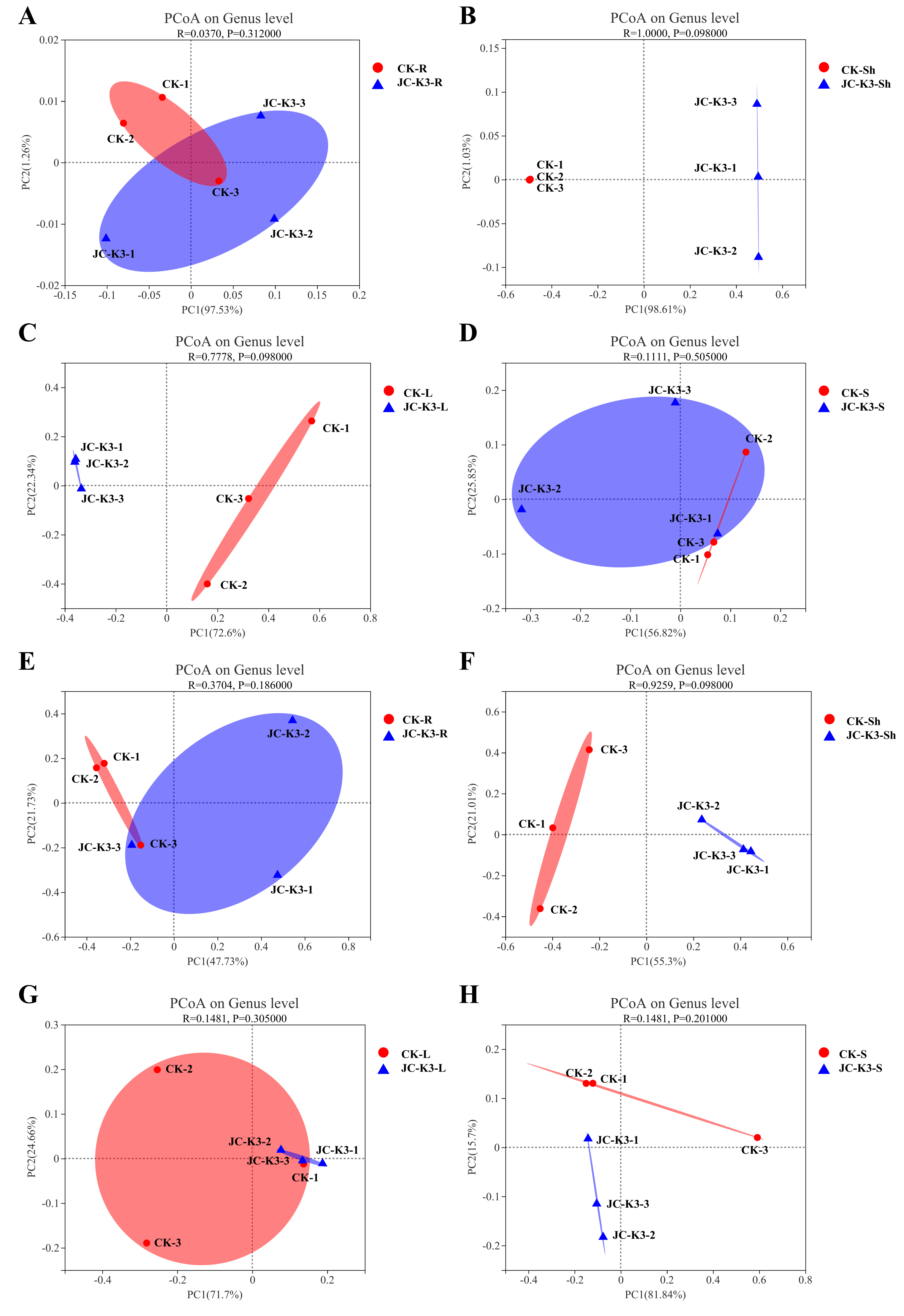
**Figure S7 | The principal co-ordinates analysis (PCoA) analysis based on Bray-Curtis distance was used to study the community composition of bacteria and fungi in plant roots, shoots, leaves and rhizosphere soils inoculated with JC-K3 strain.** (A) PCoA analysis of endophytic bacterial communities in wheat roots at genus level; (B) PCoA analysis of endophytic bacterial community in wheat stem at genus level; (C) PCoA analysis of endophytic bacterial community in wheat leaves at genus level; (D) PCoA analysis of bacterial community in wheat rhizosphere at genus level; (E) PCoA analysis of endophytic fungal communities in wheat roots at genus level; (F) PCoA analysis of endophytic fungal communities in wheat stems at genus level; (G) PCoA analysis of endophytic fungal communities in wheat leaves at genus level; (H) PCoA analysis of rhizosphere fungi in wheat at genus level. CK and JC-K3 represent the control and JC-K3 inoculated plants, respectively. S, soil; R, roots; Sh, shoots; L, leaves.


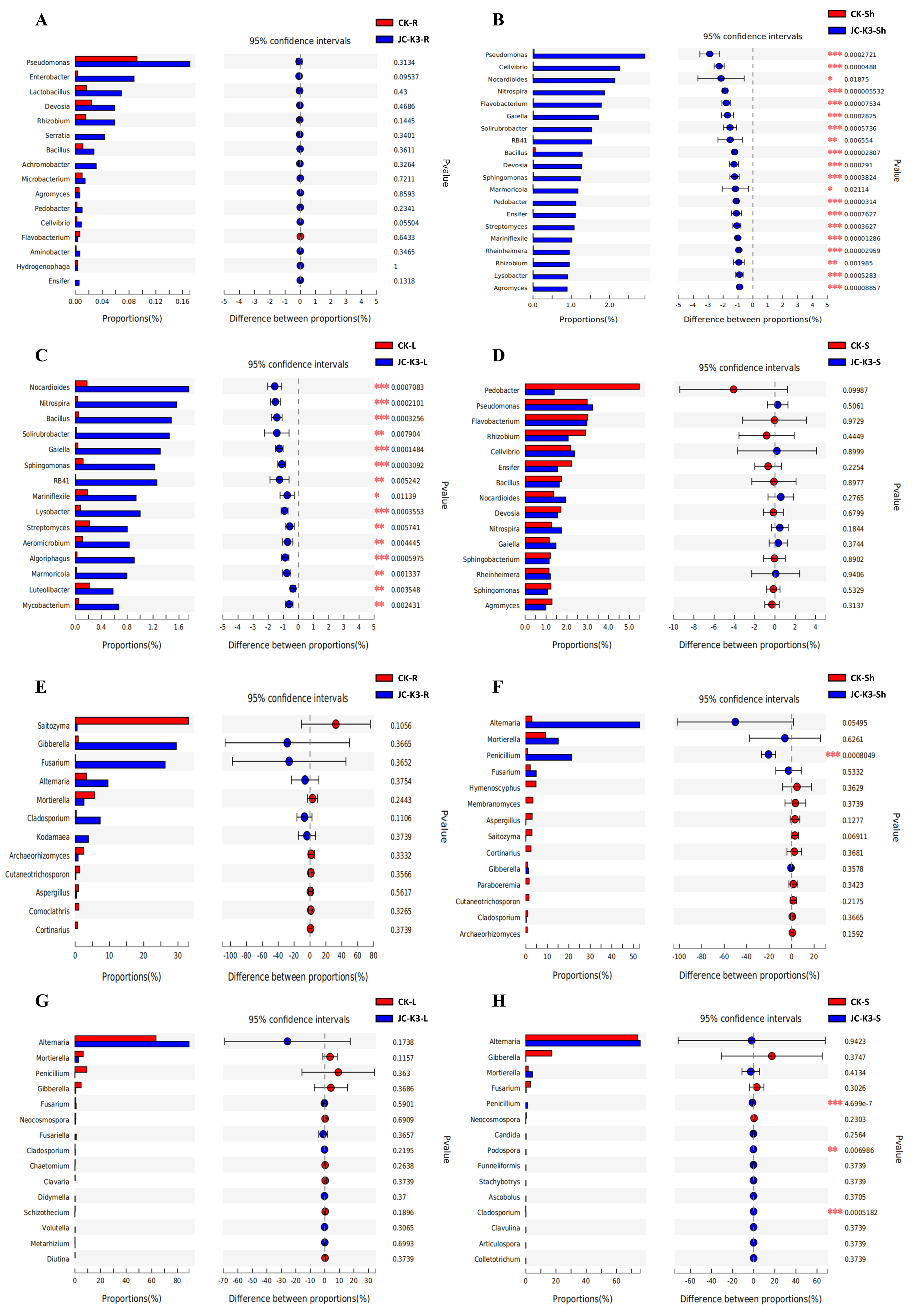
**Figure S8 | The significant differences in microbial groups at the phylum level among the root, shoot, leaf and rhizosphere soils of wheat treated with different treatments (Student's t-test bar plot on genus level).** Compared with the control group, (A) the difference of endophytic bacteria in wheat roots at genus level; (B) the difference of endophytic bacteria in wheat shoots at genus level; (C) the difference of endophytic bacteria in wheat leaves at genus level; (D) the difference of rhizosphere bacteria in wheat rhizosphere soil at genus level; (E) the difference of endophytic fungi in wheat roots at genus level; (F) the difference of endophytic fungi in wheat shoots at genus level; (G) the difference of endophytic fungi in wheat leaves at genus level; (H) the difference of rhizosphere fungi in wheat rhizosphere soil at genus level. CK and JC-K3 represent the control and JC-K3 inoculated plants, respectively. S, soil; R, roots; Sh, shoots; L, leaves. The rightmost is *P* value, *0.01< *P* ≤0.05, **0.001< *P* ≤0.01, ****P* ≤0.001.
